# Support factors for sustaining interpreters' wellbeing and professional functioning: a fuzzy Delphi study

**DOI:** 10.3389/fpubh.2026.1941746

**Published:** 2026-10-06

**Authors:** Chunwen Yang, Yanfei Li, Yijia He, Qinghe Guo

**Affiliations:** College of Foreign Languages, Tarim University, Alar, Xinjiang, China

**Keywords:** expert consensus, fuzzy Delphi method, interpreter wellbeing, occupational health, organizational support, professional functioning, work design

## Abstract

**Background:**

Interpreters work under substantial cognitive, emotional, physical, and organizational demands, yet evidence on the resources supporting their wellbeing and professional functioning remains dispersed across settings and modalities. This study developed and prioritized a multilevel support framework through expert consensus.

**Methods:**

A literature-informed framework comprising four dimensions and 31 candidate sub-themes was developed through a targeted narrative review, thematic synthesis, and content and face review. Of 20 experts invited, 15 completed an online questionnaire using a seven-point importance scale. Ratings were analyzed using the Fuzzy Delphi Method according to predefined consensus and retention criteria.

**Results:**

All four dimensions and 27 candidate sub-themes met the retention criteria, yielding a sub-theme retention rate of 87.10%. Within the expert panel, professional preparedness, agency, and sustainable competence received the strongest dimension-level endorsement (*A* = 0.838). Organizational, employment, and occupational health support ranked fourth at the dimension level (*A* = 0.693), yet contained the most strongly endorsed candidate overall, manageable workload, scheduling, breaks, recovery time, and assignment rotation (*A* = 0.902). This was followed by two D2 candidates: assignment-specific contextual and terminological preparation (*A* = 0.860) and decision latitude (*A* = 0.833). Four candidates fell below the expert-agreement criterion (60.00%−73.33%): trauma- and emotion-informed interpreting competence, inclusion as a member of the service team, confidential professional supervision, and acoustic and hearing safety. Each satisfied the mean-threshold and defuzzification criteria. They were thus excluded from the proposed cross-context core and warrant context-specific reassessment.

**Conclusion:**

The resulting panel-endorsed content framework advances a multilevel conceptualization of individual, professional, relational, and organizational resources and provides a structured basis for interpreter education, professional support, organizational policy, and occupational-health planning. While the rankings are confined to the relative priorities of this 15-member panel and provide no basis for a universal or causal hierarchy, the framework offers a foundation for subsequent measurement, validation, and implementation research involving more diverse panels across interpreting settings and modalities.

## 1 Introduction

Interpreting is a cognitively, emotionally, physically, and professionally demanding activity. Interpreters must manage real-time language processing, divided attention, rapid decision-making, and limited opportunities to correct errors. Depending on the setting, they may also encounter traumatic or emotionally charged content, role ambiguity, ethical dilemmas, complex interpersonal dynamics, and tensions surrounding professional boundaries (1–4). These demands may be compounded by hearing and vocal strain, repetitive movement, poor posture, unsuitable workspaces, technological difficulties in remote interpreting, demanding schedules, insufficient breaks, low remuneration, employment insecurity, and limited professional recognition (5–9). Interpreters' wellbeing is therefore essential to safe, effective, and sustainable practice.

Existing studies indicate that sustainable interpreting practice involves both the promotion of positive occupational outcomes and the prevention or management of adverse outcomes. Positive outcomes include job satisfaction, compassion satisfaction, professional meaning, work engagement, and career continuity, whereas adverse outcomes include occupational stress, psychological distress, emotional exhaustion, burnout, vicarious trauma, secondary traumatic stress, and turnover intentions (1, 6, 7, 10–13). Although interpreters' occupational health has received growing scholarly attention, the literature remains fragmented. Much of the research has focused on negative consequences, while the factors that positively sustain wellbeing and professional functioning have received comparatively limited systematic attention. Existing studies have proposed numerous potentially helpful resources, including self-care, emotional regulation, physical and ergonomic protection, assignment preparation, professional agency, peer support, mentoring, interdisciplinary collaboration, supervision, debriefing, psychological care, manageable workloads, fair remuneration, professional recognition, and continuing professional development (1, 5, 8, 14–17). However, these findings have not been collectively examined within a unified framework, and many recommended practices have not been systematically evaluated or prioritized. Consequently, there is no consolidated consensus framework integrating the personal, physical, professional, relational, supervisory, psychological, and organizational resources that may help interpreters sustain their wellbeing and professional functioning. A systematic process of expert judgment is therefore required to refine these factors, establish consensus regarding their relevance, and determine their relative priorities.

The Fuzzy Delphi Method (FDM) thus offers an appropriate approach for addressing this gap. FDM combines the consensus-building principles of the traditional Delphi technique with fuzzy set theory to manage the uncertainty and vagueness inherent in expert judgments (18–20). Although the conventional Delphi method is widely used to establish expert consensus on complex or insufficiently structured issues (21, 22), FDM converts experts' judgments into triangular fuzzy numbers. This enables judgments to be analyzed systematically in terms of consensus, acceptance, and priority ranking (23–25). FDM may also streamline the consultation process by reducing the number of iterative rounds required while preserving the central principle of expert consensus. FDM was therefore considered suitable for the present study since the literature offers a broad but fragmented collection of potential support factors whose conceptual relevance and relative importance have not been collectively assessed. Accordingly, the present study pursues the following research objective:

*1*. To identify and prioritize the support factors for sustaining interpreters' wellbeing and professional functioning using the FDM.

## 2 Literature review

Research on interpreters' wellbeing identifies resources across six interrelated domains: personal awareness and recovery; physical and ergonomic health protection; professional preparedness and agency; peer, collegial, and interdisciplinary support; structured supervision and psychological support; and supportive job design and organizational conditions. The following subsections summarize the evidence within each domain and provide the conceptual basis for the candidate-factor synthesis described in Section 3.2.

### 2.1 Personal awareness, emotional regulation, and recovery

Personal awareness, emotional regulation, and recovery refer to interpreters' capacity to recognize signs of occupational strain, regulate their emotional responses during assignments, and restore depleted psychological and physical resources afterward. Studies have shown that interpreters need to recognize early manifestations of overload, including persistent fatigue, intrusive recollections, sleep disruption, emotional numbing, reduced concentration, cynicism, and declining perceived effectiveness; delayed recognition may allow stress reactions to develop into burnout or secondary traumatic stress (1, 16). In addition, during demanding encounters, interpreters have reported using attentional refocusing, mindful awareness, breathing, temporary emotional distancing, and concentration on the linguistic task to remain composed and preserve communicative performance. However, distancing is not uniformly adaptive, as coping that suppresses engagement or obstructs interaction may undermine professional functioning (2, 8). Constructive recovery therefore also requires post-assignment processing through reflection, writing, confidential discussion, or professional help, together with restorative routines such as sleep, exercise, relaxation, hobbies, social connection, and deliberate separation of work from private life (5, 14, 26). Psychological detachment also becomes significant where emotionally charged material continues to occupy the interpreter after an assignment. Individual standards also matter as maladaptive perfectionism has been linked to burnout indirectly through heightened perceived stress, suggesting that interpreters benefit from maintaining high standards while responding constructively to mistakes and human limitations (27). Finally, research suggests that personal coping should include timely help-seeking when self-management is insufficient. Although resilience, flexibility, and self-care may support adaptation, they cannot sustainably compensate for unreasonable workloads, unsafe environments, or absent organizational support (5).

### 2.2 Physical and ergonomic health protection

Interpreters' wellbeing and professional functioning also depend on protection from modality-specific physical and ergonomic hazards. Spoken-language interpreters, for example, rely intensively on hearing and voice, making safe sound levels, suitable headsets, reliable audio transmission, appropriate microphone technique, hydration, vocal rest, and timely assessment of persistent auditory or vocal symptoms important preventive considerations while poor sound quality in booths or remote platforms may simultaneously increase listening effort, cognitive load, and the risk of acoustic injury (5, 9). Signed-language interpreters face a different exposure profile involving repetitive upper-limb movements, sustained or constrained postures, visual concentration, and limited opportunities for muscular recovery. A recent study identified repetitive movement, sustained posture, stress, and workplace violence as recurrent ergonomic and psychosocial risks for sign-language interpreters and guide-interpreters, with potential consequences including musculoskeletal disorders, burnout, and vicarious trauma (28). Across modalities, preventive practice therefore includes neutral and varied posture, appropriately positioned screens and equipment, adequate seating and legroom, suitable lighting, ventilation and temperature, movement and stretching, and early response to pain or repetitive-strain symptoms. Environmental design is equally consequential. For instance, permanent and portable booths, institutional work areas, and home-based remote-interpreting stations should support safe audibility, visibility, posture, and concentration rather than requiring interpreters to adapt continuously to unsuitable conditions (9).

### 2.3 Professional preparedness, agency, and boundary management

Professional preparedness, agency, and boundary management refer to the knowledge, role clarity, and professional control that enable interpreters to manage assignment demands safely and ethically. Across public-service and medical settings, studies identify inadequate preparation and uncertainty as sources of strain, while supporting contextual briefings, terminology preparation, role-play, and setting-specific education as means of strengthening readiness (1, 4, 8, 15). Such preparation becomes particularly important when competence must extend beyond linguistic transfer to include relevant technologies, interactional conventions, trauma awareness, emotional demands, and available coping resources. Accordingly, trauma- and emotion-informed education may help interpreters anticipate difficult encounters, recognize secondary-trauma reactions, and seek appropriate support rather than relying on coping strategies developed without formal guidance (3, 16). Nevertheless, preparedness alone may be insufficient when interpreters face unclear or conflicting expectations concerning neutrality, advocacy, cultural mediation, confidentiality, and emotional involvement. Role clarification and boundary management are therefore necessary for defining interpreters' responsibilities within complex interactions (1, 2). Moreover, these boundaries need to be accompanied by professional agency, including the discretion to request clarification, negotiate conditions, pause, seek assistance, or decline assignments that may compromise safety or quality. Schwenke's (29) identification of decision latitude as the strongest work-related predictor of emotional exhaustion further suggests that preparation, role clarity, and professional control operate together as protective resources.

### 2.4 Peer, collegial, and interdisciplinary support

Peer, collegial, and interdisciplinary support refers to professional relationships that provide emotional understanding, practical assistance, shared learning, and coordinated responses to demanding assignments. In refugee, community, and medical interpreting, peer exchange has been consistently identified as a resource for discussing difficult experiences, normalizing emotional reactions, obtaining practical advice, and reducing professional isolation (1, 5, 8). In addition to emotional support, interpreter teams may redistribute demanding assignments, provide temporary coverage, and draw on more experienced colleagues when cases exceed an individual interpreter's current capacity. Mentoring extends this collegial function by supporting novice interpreters' transition into practice, professional judgment, and gradual exposure to complex assignments (14). However, support within the interpreting profession represents only one part of the relational environment. As interpreting is embedded in interactions with clinicians, counselors, legal professionals, and other service providers, interdisciplinary inclusion is also important for ensuring access to relevant information, clarifying responsibilities, and coordinating difficult encounters. Evidence from medical and palliative-care settings indicates that clinician-interpreter dialogue, collaborative briefing, and recognition of interpreters as team members can strengthen preparation, mutual respect, ethical discussion, and emotional support (15, 30). Similarly, collaborative responses appear more conducive to effective management of emotionally charged interactions than coping strategies that obstruct communication (2).

### 2.5 Structured supervision and psychological support

Structured supervision and psychological support refer to formal resources for professional reflection, post-assignment processing, and specialized care when occupational strain exceeds personal or collegial coping capacities. Studies indicate a persistent mismatch between interpreters' exposure to traumatic or emotionally demanding material and their access to formal support, particularly in refugee and mental-health settings (1). Within this support continuum, professional supervision provides ongoing reflection on emotional responses, ethical tensions, role conflicts, and practice-related difficulties, whereas post-assignment debriefing facilitates case-specific processing and closure. Psychological counseling and trauma-related care serve a further function by addressing distress that requires individualized professional intervention. Evidence from medical interpreting supports the value of these resources but also reveals that interpreters have requested structured debriefing and support groups and valued access to trauma-informed professionals, yet contractual status, scheduling, service location, and exclusion from clinical-team debriefings may restrict participation (8, 15). The need for accessible support is reinforced by the high prevalence of secondary traumatic stress reported among interpreters working with refugees, including the finding that 67% exhibited at least mild symptoms (16).

### 2.6 Supportive job design, organizational culture, and career sustainability

Supportive job design, organizational culture, and career sustainability refer to employment and institutional resources that limit excessive demands while enabling recognition, development, and continued professional participation. Occupational strain is linked to high work intensity, repeated exposure to distressing assignments, inadequate breaks, unpredictable scheduling, payment dissatisfaction, insecure employment, and insufficient professional recognition (1, 5, 7, 8). Hanft-Robert and Mösko (31) also found that 36% of community interpreters reported moderate or severe interpreting-related distress and that higher distress was associated with greater interpreting frequency and lower payment satisfaction. Accordingly, sustainable job design requires manageable workloads, adequate breaks, assignment rotation, planning certainty, fair remuneration, and payment for preparation and other related professional activities. Beyond workload and compensation, organizational culture determines whether interpreters are respected, included, and able to report overload, errors, discrimination, abusive conduct, or unsafe conditions without stigma or retaliation. This concern is particularly evident in remote settings, where video-relay interpreters reported abusive caller behavior but limited reporting and inadequate organizational responses (32). Moreover, access to training, supervision, psychological support, and other organizational resources may vary according to employment status, leaving freelance and contract interpreters comparatively unprotected (8). Over time, occupational stress may increase turnover intentions through burnout, whereas supportive environments, continuing development, professional meaning, and compassion satisfaction may strengthen retention (6, 13). Organizational support is therefore a central condition of sustainable practice.

## 3 Materials and methods

### 3.1 Research design

This study employed a consensus-based framework-development and prioritization design using the FDM to evaluate, refine, and prioritize the support factors considered important for sustaining interpreters' wellbeing and professional functioning. Rather than testing relationships among predetermined variables, the present study sought to establish an expert-informed consensus framework for a field in which relevant practices and resources remain dispersed across different interpreting settings, modalities, and occupational-health perspectives. This design was therefore appropriate for consolidating the existing evidence into a structured set of dimensions and candidate sub-themes and subjecting them to systematic expert appraisal.

FDM integrates the consensus-oriented logic of the conventional Delphi technique with fuzzy set theory, allowing imprecise or subjective expert judgments to be represented and analyzed quantitatively (18–20). In a traditional Delphi process, experts evaluate a complex issue through successive rounds until an acceptable level of agreement is achieved (21, 22). FDM extends this procedure by transforming importance ratings into triangular fuzzy numbers, thereby enabling the assessment of item-level consensus, acceptance, and relative priority (23–25). It may also reduce the need for repeated consultation rounds while retaining the central principle of collective expert judgment. This feature was particularly relevant to the present study because the literature identifies a broad range of potentially beneficial personal, professional, relational, psychological, physical, and organizational resources, but their relative importance has not been established through expert consensus.

The study was conducted in three sequential stages (see Figure 1). In the first stage, relevant empirical and conceptual literature was reviewed and thematically synthesized to construct a preliminary framework comprising four dimensions and 31 candidate sub-themes. In the second stage, an FDM questionnaire was developed and reviewed before being distributed to a panel of eligible experts, who assessed the importance of each dimension and sub-theme to interpreters' wellbeing and professional functioning. In the third stage, the expert ratings were converted into triangular fuzzy numbers and analyzed using threshold values, percentage agreement, defuzzification scores, and priority rankings. The retention or non-retention of each candidate sub-theme was determined according to the predefined FDM decision criteria, after which the framework was revised to produce the final consensus-based structure.

**Figure 1:**
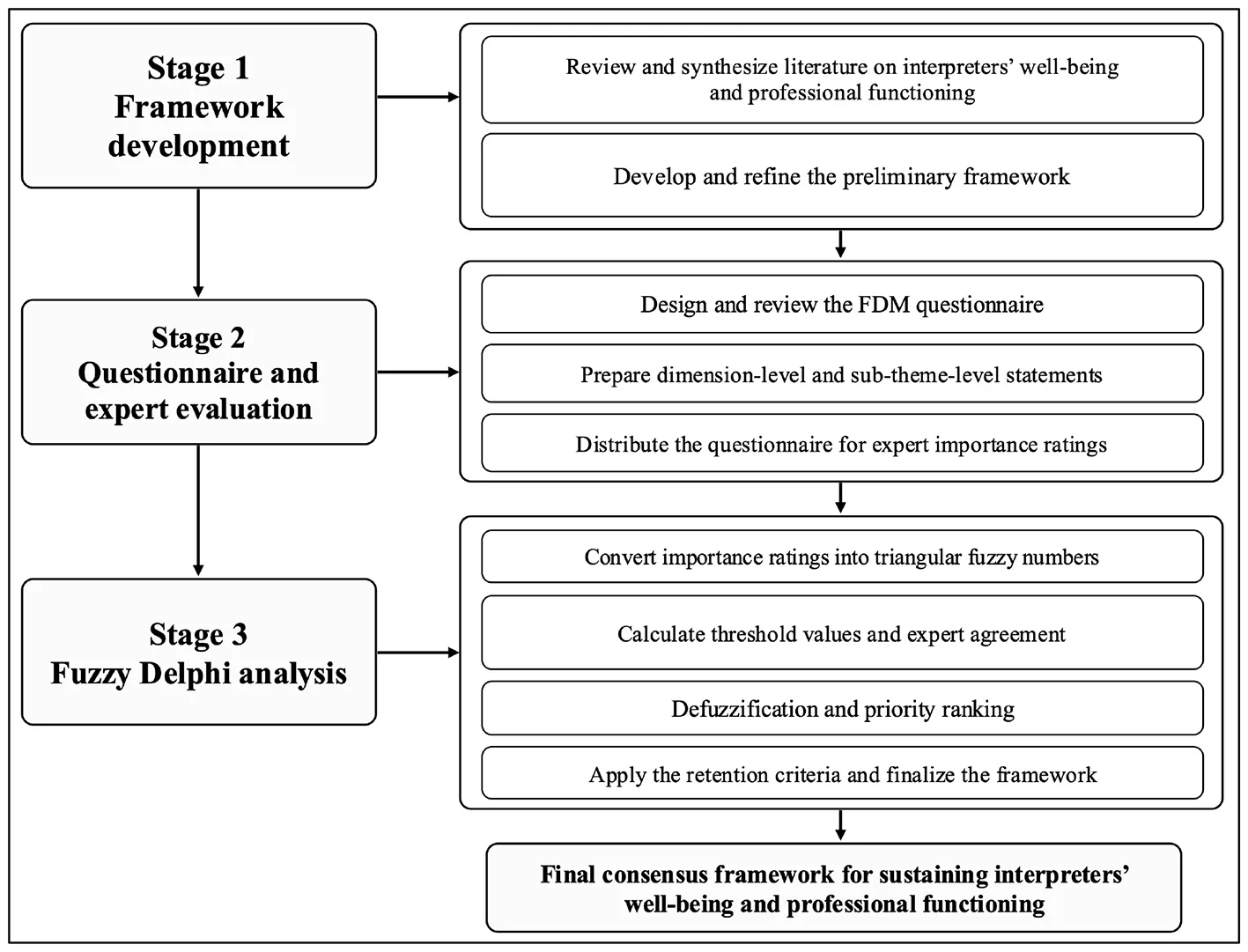
Research procedure of the present FDM study [adapted from Al-Rikabi and Montazer (49)].

### 3.2 Development and refinement of the initial framework

The initial framework was developed through a targeted narrative literature review followed by a structured thematic synthesis. The review aimed to identify practices, competencies, relational resources, working conditions, and organizational provisions associated with interpreters' wellbeing and professional functioning. It served as an evidence-informed candidate-generation process for subsequent content review and expert appraisal.

Electronic searches were conducted through Google Scholar, with no lower publication-date limit, and were completed on 15 April 2026. The searches were supplemented by backward searching of the reference lists of relevant publications and citation searching. The search strategy combined terms describing the interpreter population with terms related to occupational health, wellbeing, and support. Population terms included *interpreter, spoken-language interpreter, sign-language interpreter, community interpreter, public-service interpreter, medical interpreter, healthcare interpreter, refugee interpreter, conference interpreter, simultaneous interpreter, remote interpreter*, and *video-mediated interpreter*. Occupational-health and support terms included *wellbeing, wellness, occupational health, occupational stress, burnout, emotional exhaustion, psychological distress, vicarious trauma, secondary traumatic stress, resilience, self-care, coping, recovery, psychological detachment, workload, ergonomics, hearing safety, vocal health, supervision, debriefing, peer support, mentoring, professional agency, decision latitude, role clarity, remuneration, employment security, professional recognition, professional development*, and *turnover intentions*. The search terms were adjusted when relevant terminology emerged across different interpreting settings and modalities.

English-language publications were eligible when they provided extractable evidence concerning interpreters' psychological, emotional, physical, professional, relational, or occupational wellbeing. Eligible sources comprised qualitative, quantitative, and mixed-methods studies, scholarly reviews, book chapters, and scholarly volumes covering spoken-language and signed-language interpreting in community, public-service, healthcare, refugee, conference, simultaneous, remote, and video-mediated settings. Studies involving translators were considered when they examined occupational mechanisms directly transferable to interpreting, including remuneration, employment insecurity, agency relationships, and career sustainability. Two general occupational-health publications were also eligible when they supplied a directly relevant theoretical framework or construct definition used to organize or define the candidate factors. Publications focused exclusively on linguistic performance, interpreting accuracy, pedagogy, technology performance, or service-user outcomes were excluded when they provided no extractable support factor. Duplicate records, non-English publications, and sources with insufficient substantive information were also excluded. The final framework-development corpus comprised 26 source documents published between 2007 and 2026. Their characteristics and documented contributions are presented in Supplementary Table S1.

The first author conducted the literature search, reviewed the eligible publications, and extracted practices, resources, competencies, and working conditions relevant to interpreters' wellbeing and professional functioning. A publication could contribute more than one factor when it addressed several relevant practices or occupational mechanisms. The literature links for the 31 refined candidate sub-themes are documented in Supplementary Table S2. For the intercoder reliability assessment, the first and second authors independently classified the 31 extracted factors within the six provisional domains.

First-cycle coding assigned descriptive labels that preserved the substantive meaning of the evidence, such as emotional self-regulation, restorative recovery, assignment preparation, decision latitude, peer consultation, professional supervision, manageable workload, ergonomic safety, fair remuneration, and professional recognition. During second-cycle coding, the labels were compared across publications according to semantic similarity, protective mechanism, provider, timing, structure, and ecological locus. Codes describing the same mechanism at the same level of action were merged. Codes with different providers, timing, formalization, or loci of responsibility were retained separately. For example, individual emotional processing was distinguished from formal post-assignment debriefing, peer consultation from professional supervision, and supervision from qualified psychological care.

This process generated 31 candidate factors across six provisional descriptive domains: (1) personal awareness, emotional regulation, and recovery; (2) physical and ergonomic health protection; (3) professional preparedness, agency, and boundary management; (4) peer, collegial, and interdisciplinary support; (5) structured supervision and psychological support; and (6) supportive job design, organizational culture, and career sustainability. The six domains contained seven, four, five, four, three, and eight candidate factors, respectively. Higher-order axial coding consolidated them into four dimensions according to their primary ecological locus and support mechanism. The personal domain formed the individual dimension, and professional preparedness and agency formed the professional dimension. Peer and interdisciplinary support was combined with structured supervision and psychological care to form the relational and formal-support dimension. Physical and ergonomic protection was combined with job design and organizational conditions to form the organizational and occupational-conditions dimension. This consolidation preserved all 31 candidate factors while reducing overlap among the higher-order categories.

Agreement between the two independent classifications was assessed using Cohen's kappa and reached κ = 0.89. Differences mainly concerned the conceptual boundaries between closely related factors and their placement within the six provisional domains. The authors resolved these differences through discussion, re-examination of the relevant literature, and application of the agreed coding definitions. Following consensus coding and higher-order consolidation, the resulting four dimensions and 31 candidate sub-themes formed the draft framework presented in Table 1.

**Table 1:** Draft framework for sustaining interpreters' wellbeing and professional functioning before expert review.

Dimensions	Candidate sub-themes
D1. Individual awareness, regulation, and recovery	ST1. Recognition of occupational strain and early warning signs
	ST2. Emotional self-regulation during interpreting assignments
	ST3. Constructive emotional processing after difficult assignments
	ST4. Restorative physical and psychological self-care routines
	ST5. Psychological detachment from work during non-work time
	ST6. Adaptive professional standards and constructive responses to mistakes
	ST7. Timely help-seeking when personal coping is insufficient
D2. Professional preparedness, agency, and boundary management	ST8. Assignment-specific contextual and terminological preparation
	ST9. Competence for the interpreting setting and modality
	ST10. Trauma-informed and emotionally informed interpreting competence
	ST11. Clarity of the interpreter's professional role and ethical boundaries
	ST12. Professional decision latitude in managing assignment demands
D3. Relational and formal support systems	ST13. Peer consultation and mutual support among interpreters
	ST14. Professional mentoring by experienced interpreters
	ST15. Inclusion of interpreters as members of the service team
	ST16. Collaborative pre-assignment briefing and case coordination
	ST17. Routine confidential professional supervision
	ST18. Structured post-assignment debriefing and case reflection
	ST19. Access to professional counseling and trauma-related psychological care
D4. Organizational conditions, occupational health protection, and career sustainability	ST20. Sustainable workload, scheduling, breaks, and assignment rotation
	ST21. Hearing protection and acoustic safety
	ST22. Vocal health protection
	ST23. Musculoskeletal and repetitive-strain prevention
	ST24. Ergonomically and environmentally safe interpreting workspaces
	ST25. Fair remuneration for interpreting and related professional time
	ST26. Employment security and fair contracting conditions
	ST27. Professional recognition, respect, and status
	ST28. Psychologically safe reporting of overload, errors, and health concerns
	ST29. Equitable, paid, and protected access to organizational support resources
	ST30. Continuing professional development across the career span
	ST31. Professional meaning, compassion satisfaction, and career continuity

Before questionnaire construction, the draft framework underwent content and face review by four reviewers: two experienced professional interpreters and two researchers with expertise in interpreting studies, occupational wellbeing, or research methodology. The professional interpreters assessed practical relevance, clarity, feasibility, and applicability across settings. The researchers assessed conceptual clarity, internal consistency, coverage, domain placement, and overlap. They identified ambiguous or compound wording, overlapping concepts, misplaced factors, and potentially omitted forms of support.

The review retained the four-dimensional architecture and refined the candidate sub-themes. Within D1, wording was clarified to distinguish individual emotional processing, physical recovery, psychological detachment, and constructive management of mistakes. D2 was renamed to incorporate sustainable competence; continuing professional development was moved into this dimension, and role clarity, trauma-informed competence, and decision latitude were specified more precisely. D3 was renamed to reflect collegial, interdisciplinary, and professional support, with clearer boundaries among peer support, mentoring, team inclusion, collaborative briefing, supervision, debriefing, and psychological care. Within D4, vocal and musculoskeletal protection were merged, reliable technology and technical support were added, and the workload, remuneration, reporting, and organizational-support factors were clarified. Organizational protection from abusive, threatening, or violent conduct was added, while professional meaning, compassion satisfaction, and career continuity were classified as potential outcomes of the framework and removed from the candidate-factor set. A detailed comparison of the draft and refined preliminary frameworks is provided in Supplementary Table S3.

Following these revisions, the refined preliminary framework comprised four dimensions and 31 candidate sub-themes, as presented in Table 2. The revisions clarified the wording, domain placement, and conceptual boundaries of the candidate sub-themes while preserving the overall size of the framework. Supplementary Table S4 provides the operational definitions and selected supporting-evidence codes for the refined candidates. The four dimensions and 31 candidate sub-themes were then converted into questionnaire statements for expert evaluation through FDM.

**Table 2:** Refined preliminary framework for sustaining interpreters' wellbeing and professional functioning.

Dimensions	Candidate sub-themes
D1. Individual awareness, regulation, and recovery	ST1. Recognition of occupational strain and early warning signs
	ST2. Emotional self-regulation during assignments
	ST3. Individual reflection and emotional processing after difficult assignments
	ST4. Physical recovery and restorative self-care
	ST5. Psychological detachment and work–life boundaries
	ST6_Realistic professional standards and constructive management of mistakes
	ST7_Timely help-seeking when personal coping is insufficient
D2. Professional preparedness, agency, and sustainable competence	ST8. Assignment-specific contextual and terminological preparation
	ST9. Setting- and modality-specific professional competence
	ST10. Trauma- and emotion-informed interpreting competence
	ST11. Role clarity and ethical boundary management
	ST12. Decision latitude, including the ability to pause, seek assistance, or decline unsafe conditions
	ST13. Continuing professional development across the career span
D3. Collegial, interdisciplinary, and professional support	ST14. Peer consultation and mutual support
	ST15. Professional mentoring
	ST16. Inclusion as a member of the service team
	ST17. Collaborative briefing and case coordination
	ST18. Confidential professional supervision
	ST19. Formal post-assignment debriefing
	ST20. Access to qualified psychological and trauma-related care
D4. Organizational, employment, and occupational health support	ST21. Manageable workload, scheduling, breaks, recovery time, and assignment rotation
	ST22. Acoustic and hearing safety
	ST23. Vocal and musculoskeletal health protection
	ST24. Ergonomically and environmentally safe workspaces
	ST25. Access to reliable technology and technical support
	ST26. Fair remuneration, including preparation and related professional time
	ST27. Employment security and fair contracting
	ST28. Professional recognition, respect, and status
	ST29. Safe reporting and effective responses to overload, adverse incidents, discrimination, and health concerns
	ST30. Protected and remunerated access to organizational support resources
	ST31. Organizational protection from abusive, threatening, or violent conduct

### 3.3 FDM questionnaire

The FDM questionnaire was developed from the refined preliminary framework presented in Table 2. It included a background-information section and two substantive rating sections. The background-information section collected the demographic and professional characteristics reported in Table 3. The first rating section contained four statements corresponding to the four overarching dimensions, while the second contained 31 statements corresponding to the candidate sub-themes within those dimensions. This structure enabled separate evaluation of the overall framework and its individual components. The questionnaire items and response format are provided in Supplementary Table S5.

**Table 3:** Demographic characteristics of the expert panel.

Expert	Gender	Age	Highest academic qualification	Professional role	Relevant professional experience
1	Male	39	Master's degree	Practicing interpreter	11–20 years
2	Male	37	Master's degree	Practicing interpreter	11–20 years
3	Female	40	Doctoral degree	Interpreting researcher	11–20 years
4	Female	34	Master's degree	Manager of interpreting services	≤ 10 years
5	Male	36	Master's degree	Practicing interpreter	11–20 years
6	Female	44	Doctoral degree	Interpreting educator (Professor)	11–20 years
7	Female	35	Master's degree	Interpreting educator (Lecturer)	≤ 10 years
8	Male	38	Master's degree	Interpreting educator (Lecturer)	11–20 years
9	Female	42	Master's degree	Interpreting trainer (Senior Lecturer)	11–20 years
10	Female	40	Master's degree	Interpreting educator (Associate Professor)	11–20 years
11	Female	45	Doctoral degree	Interpreting educator (Professor)	11–20 years
12	Female	37	Master's degree	Practicing interpreter	11–20 years
13	Male	46	Master's degree	Interpreting trainer (Senior lecturer)	21–30 years
14	Male	40	Master's degree	Interpreting educator (Professor)	11–20 years
15	Male	39	Doctoral degree	Interpreting educator (Associate professor)	11–20 years

Experts rated the importance of each dimension and candidate sub-theme for sustaining interpreters' wellbeing and professional functioning. A seven-point importance scale was used, ranging from 1 (extremely unimportant) to 7 (extremely important). The questionnaire provided completion instructions and an explanation of the response scale. Experts were asked to base their ratings on their professional knowledge and experience.

### 3.4 Expert panel, recruitment, and data collection

Purposive sampling was used to recruit experts with knowledge and experience relevant to the study. Expert selection in Delphi research emphasizes participants' capacity to provide informed judgments and their familiarity with the topic under investigation (33, 34). Eligibility required at least a master's degree, a minimum of five years of relevant professional experience, and current or recent involvement in interpreting practice, interpreter education or training, interpreting research, or the management of interpreting services. Eligible participants were also required to be familiar with interpreters' occupational demands and support needs and willing to complete the expert evaluation.

Published recommendations concerning Delphi panel size vary according to the topic and the level of expertise required. Rowe and Wright (22) suggested panels of approximately 5–20 experts, while Okoli and Pawlowski (21) recommended 10–18 experts. Panels of approximately 10–15 experts have also been considered sufficient when participants possess closely relevant expertise (35, 36). Panel composition was therefore determined by the relevance of participants' expertise, professional diversity, experience, and willingness to participate (24, 37–41). Based on these criteria, 20 eligible experts were invited to participate.

The 20 experts were contacted individually by email. The invitation introduced the purpose of the study, explained the basis for the recipient's selection, described the expected task, and provided information on voluntary participation and the confidential treatment of responses. Fifteen experts agreed to participate and received a link to the online questionnaire hosted on Wenjuanxing, a web-based survey platform. Before completing the questionnaire, participants reviewed the study information and provided electronic informed consent. All 15 experts opened or began the questionnaire, submitted it, and completed ratings for all four dimensions and all 31 candidate sub-themes. No questionnaire was excluded for incomplete responses. The final analytic sample therefore comprised 15 experts, representing 75.0% of the 20 experts invited. Data collection was conducted from 5 May to 16 May 2026.

The final panel incorporated practitioner and academic perspectives. It comprised nine interpreter educators or trainers (60.0%), four practicing interpreters (26.7%), one interpreting researcher (6.7%), and one manager of interpreting services (6.7%). Participants were aged 34–46 years, with a mean age of 39.47 years. Eight were female (53.3%), and seven were male (46.7%). Eleven held master's degrees (73.3%), and four held doctoral degrees (26.7%). Regarding relevant professional experience, 12 experts reported 11–20 years of experience (80.0%), two reported 10 years or less (13.3%), and one reported 21–30 years (6.7%). Table 3 presents the individual characteristics of the expert panel.

### 3.5 FDM analysis

The expert ratings were analyzed using FDM. Responses at the dimension level and sub-theme level were processed separately so that consensus could be examined for both the overall framework structure and its individual components. The analysis involved four steps: fuzzification of the importance ratings, calculation of consensus indicators, defuzzification, and priority ranking. All FDM calculations were performed using Microsoft Excel for Mac (Microsoft 365).

First, the seven verbal response categories on the importance scale were treated as linguistic variables and mapped to triangular fuzzy numbers. In this context, a linguistic variable refers to the importance category whose meaning is represented by a range of numerical values. A triangular fuzzy number is expressed as (*n*_1_*, n*_2_*, n*_3_), where *n*_1_ represents the lower value, *n*_2_ the most representative value, and *n*_3_ the upper value associated with an expert's judgment. Mapping each verbal response category to a triangular fuzzy number represents the imprecision associated with expert judgments and permits the fuzzy analysis of consensus and priority (18, 20). The seven-point importance scale and corresponding triangular fuzzy numbers used in the present study are shown in Table 4. Higher fuzzy values indicate stronger endorsement of a dimension or sub-theme.

**Table 4:** Seven-point importance scale and corresponding triangular fuzzy numbers.

Scale	Importance category	Triangular fuzzy number (*n_1_, n_2_, n_3_*)
1	Extremely unimportant	(0.00, 0.00, 0.10)
2	Unimportant	(0.00, 0.10, 0.30)
3	Somewhat unimportant	(0.10, 0.30, 0.50)
4	Neutral	(0.30, 0.50, 0.70)
5	Somewhat important	(0.50, 0.70, 0.90)
6	Important	(0.70, 0.90, 1.00)
7	Extremely important	(0.90, 1.00, 1.00)

For each dimension and sub-theme, the fuzzy responses of the 15 experts were aggregated component by component to obtain an average fuzzy number, expressed as (*m*_1_*, m*_2_*, m*_3_). The aggregation was calculated using Equation 1:


$$\begin{array}{l} m_{1}=\frac{1}{k}\overset{k}{\underset{i=1}{\sum }}n_{1i},\;m_{2}=\frac{1}{k}\overset{k}{\underset{i=1}{\sum }}n_{2i},\;m_{3}=\frac{1}{k}\overset{k}{\underset{i=1}{\sum }}n_{3i} \end{array}$$ (1)

where *k* denotes the number of experts, and (*n*_1*i*_*, n*_2*i*_*, n*_3*i*_) represents the triangular fuzzy number assigned by expert *i*. The resulting average fuzzy number represented the collective judgment of the panel for each item (42, 43).

Second, consensus was assessed using the individual threshold value, the mean item threshold value, and the percentage of expert agreement. For each expert–item pair, the individual threshold value *d*_*i*_ was calculated using Equation 2 as the distance between the expert's fuzzy rating and the corresponding group average:


$$\begin{array}{l} d_{i}=\sqrt{\frac{1}{3}\left[{\left(m_{1}-n_{1i}\right)}^{2}+{\left(m_{2}-n_{2i}\right)}^{2}+{\left(m_{3}-n_{3i}\right)}^{2}\right]} \end{array}$$ (2)

where (*m*_1_*, m*_2_*, m*_3_) represents the aggregated fuzzy number for the item and (*n*_1*i*_*, n*_2*i*_*, n*_3*i*_) represents the fuzzy number assigned by expert _*i*_. An individual threshold value of *d*_*i*_ ≤ 0.20 indicated that the expert's judgment was sufficiently close to the collective assessment (42–44). The mean threshold value for each dimension or sub-theme was subsequently calculated using Equation 3:


$$d=\frac{1}{k}\sum_{i=1}^{k}d_{i}$$ (3)

where *k* represents the number of experts. An item satisfied the mean-threshold criterion when *d* ≤ 0.20. Expert agreement was calculated using Equation 4 as the percentage of experts whose individual threshold values satisfied *d*_*i*_ ≤ 0.20:


$$\text{Expert agreement }(\%)\text{=}\frac{\text{Number of experts with }{\text{d}}_{\text{i}}\leq \text{0}.\text{20}}{\text{Total number of experts}}\times \text{100}$$ (4)

An item was considered to have achieved adequate group consensus when at least 75% of the experts recorded individual threshold values of *d*_*i*_ ≤ 0.20 (44, 45). With a panel of 15 experts, this criterion required at least 12 experts to fall within the accepted threshold.

Third, defuzzification was conducted to convert each aggregated triangular fuzzy number into a single scalar value representing the overall level of endorsement. The defuzzified value A was calculated using the center-of-area formula shown in Equation 5:


$$\begin{array}{l} A=\frac{m_{1}+m_{2}+m_{3}}{3} \end{array}$$ (5)

The value of *A* ranges from 0 to 1, with higher values indicating stronger perceived importance. A dimension or sub-theme satisfied the importance criterion when its defuzzified value was *A* ≥ 0.50 (45, 46). The final retention decision was based on three criteria applied jointly. A dimension or sub-theme was retained when: (a) its mean threshold value satisfied *d* ≤ 0.20; (b) at least 75% of the experts recorded individual threshold values of *d*_*i*_ ≤ 0.20; and (c) its defuzzified value satisfied *A* ≥ 0.50. Items that failed to meet one or more of these criteria were not retained in the final framework.

Finally, the retained dimensions and sub-themes were prioritized according to their defuzzified values. The retained dimension with the highest *A* value received the highest dimension-level rank. Sub-theme rankings were calculated separately within each dimension, with higher *A* values indicating higher priority. Rankings were determined using unrounded *A* values, and ties were assigned only when the unrounded values were identical. All fuzzy values, consensus indicators, retention decisions, and priority rankings were calculated using unrounded values; the values presented in the tables were rounded to three decimal places for reporting. The analytical decision rules are summarized in Table 5.

**Table 5:** Decision rules for the FDM analysis.

Analytical criterion	Acceptance rule	Interpretation
Mean item threshold value	*d* ≤ 0.20	The average distance between the experts' judgments and the group judgment is acceptable
Expert agreement	≥ 75%	The item has achieved adequate panel consensus
Defuzzified value	*A* ≥ 0.50	The item met the predefined defuzzification criterion for retention
Priority ranking	Higher *A* value	The item receives a higher priority within the relevant level of analysis

## 4 Results

### 4.1 Dimension-level consensus and priority ranking

The FDM analysis first examined expert consensus and priority at the dimension level. As shown in Table 6, mean threshold values ranged from 0.081 to 0.101, expert agreement ranged from 80.00% to 100.00%, and defuzzified values ranged from 0.693 to 0.838. All four dimensions therefore satisfied the predefined retention criteria of a mean threshold value of *d* ≤ 0.20, expert agreement of at least 75%, and a defuzzified value of *A* ≥ 0.50 and were retained in the final framework.

**Table 6:** Consensus and priority ranking of the framework dimensions.

Dimension	Consensus indicators		Aggregated triangular fuzzy number			Defuzzification		
	Mean threshold value (*d* ≤ 0.20)	Expert agreement (% ≥75%)	*m*_1_	*m*_2_	*m*_3_	Defuzzified value (*A ≥*0.50)	Decision	Rank
D1	0.086	100.00%	0.593	0.793	0.947	0.778	Retained	2
D2	0.101	100.00%	0.687	0.860	0.967	0.838	Retained	1
D3	0.092	80.00%	0.513	0.707	0.880	0.700	Retained	3
D4	0.081	100.00%	0.500	0.700	0.880	0.693	Retained	4

Among the four dimensions, D2 (professional preparedness, agency, and sustainable competence) received the highest priority (*A* = 0.838). D1 (individual awareness, regulation, and recovery) ranked second (*A* = 0.778), followed by D3 (collegial, interdisciplinary, and professional support) (*A* = 0.700) and D4 (organizational, employment, and occupational health support) (*A* = 0.693). D3 recorded the lowest expert agreement at 80.00%, which remained above the required 75% criterion.

### 4.2 Sub-theme-level consensus and priority ranking

Table 7 presents the consensus indicators, aggregated triangular fuzzy numbers, retention decisions, and within-dimension priority rankings for the 31 candidate sub-themes. All 31 sub-themes satisfied the mean-threshold criterion of *d* ≤ 0.20 and the defuzzification criterion of *A* ≥ 0.50. Twenty-seven sub-themes also achieved the required expert agreement of at least 75% and were retained, producing an overall sub-theme retention rate of 87.10%. Four sub-themes fell below the expert-agreement requirement and were excluded. Rankings were assigned separately within each dimension using unrounded defuzzified values and were limited to the retained sub-themes.

**Table 7:** Sub-theme-level consensus, retention decisions, and within-dimension priority rankings.

Sub-theme	Consensus indicators		Aggregated triangular fuzzy number			Defuzzification		
	Mean threshold value ($\bar{d}$ ≤ 0.20)	Expert agreement (% ≥75%)	*m*_1_	*m*_2_	*m*_3_	Defuzzified value (*A ≥*0.50)	Decision	Rank
D1								
ST1	0.138	80.00%	0.593	0.787	0.920	0.767	Retained	2
ST2	0.097	86.67%	0.633	0.833	0.953	0.807	Retained	1
ST3	0.068	100.00%	0.553	0.753	0.927	0.744	Retained	3
ST4	0.116	86.67%	0.513	0.707	0.873	0.698	Retained	4
ST5	0.028	100.00%	0.500	0.700	0.893	0.698	Retained	4
ST6	0.096	100.00%	0.420	0.620	0.820	0.620	Retained	7
ST7	0.055	100.00%	0.500	0.700	0.887	0.696	Retained	6
D2								
ST8	0.076	100.00%	0.713	0.887	0.980	0.860	Retained	1
ST9	0.113	80.00%	0.593	0.780	0.927	0.767	Retained	4
ST10	0.165	60.00%	0.433	0.633	0.813	0.627	Not retained	-
ST11	0.095	100.00%	0.673	0.853	0.967	0.831	Retained	3
ST12	0.055	100.00%	0.660	0.860	0.980	0.833	Retained	2
ST13	0.099	93.33%	0.580	0.780	0.933	0.764	Retained	5
D3								
ST14	0.076	93.33%	0.647	0.847	0.967	0.820	Retained	1
ST15	0.123	86.67%	0.407	0.607	0.793	0.602	Retained	4
ST16	0.149	73.33%	0.393	0.593	0.780	0.589	Not retained	-
ST17	0.112	86.67%	0.607	0.807	0.940	0.784	Retained	2
ST18	0.142	73.33%	0.353	0.553	0.747	0.551	Not retained	-
ST19	0.131	80.00%	0.367	0.567	0.760	0.564	Retained	5
ST20	0.106	100.00%	0.500	0.700	0.873	0.691	Retained	3
D4								
ST21	0.075	93.33%	0.780	0.933	0.993	0.902	Retained	1
ST22	0.155	66.67%	0.367	0.567	0.753	0.562	Not retained	-
ST23	0.075	86.67%	0.313	0.513	0.713	0.513	Retained	9
ST24	0.074	100.00%	0.487	0.687	0.873	0.682	Retained	4
ST25	0.098	100.00%	0.487	0.687	0.867	0.680	Retained	5
ST26	0.107	86.67%	0.593	0.787	0.933	0.771	Retained	3
ST27	0.145	86.67%	0.487	0.680	0.847	0.671	Retained	6
ST28	0.132	80.00%	0.447	0.647	0.827	0.640	Retained	7
ST29	0.121	100.00%	0.647	0.820	0.947	0.804	Retained	2
ST30	0.160	100.00%	0.300	0.500	0.700	0.500	Retained	10
ST31	0.112	93.33%	0.447	0.640	0.827	0.638	Retained	8

Rankings were calculated separately within each dimension using unrounded defuzzified values. A dash indicates that the sub-theme failed to satisfy all three retention criteria and was excluded from the final framework.

Within D1, all seven sub-themes satisfied the three retention criteria. ST2, emotional self-regulation during assignments, received the highest priority (*A* = 0.807), followed by ST1, recognition of occupational strain and early warning signs (*A* = 0.767), and ST3, individual reflection and emotional processing after difficult assignments (*A* = 0.744). ST4, physical recovery and restorative self-care, and ST5, psychological detachment and work-life boundaries, shared fourth place (*A* = 0.698). ST7, timely help-seeking when personal coping is insufficient, ranked sixth (*A* = 0.696), while ST6, realistic professional standards and constructive management of mistakes, ranked seventh (*A* = 0.620). The complete retention of D1 indicates strong panel endorsement of individual awareness, emotional regulation, recovery, boundary management, and help-seeking as components of the proposed support framework.

Within D2, five of the six candidate sub-themes were retained. ST8, assignment-specific contextual and terminological preparation, received the highest priority (*A* = 0.860). It was followed by ST12, decision latitude, including the ability to pause, seek assistance, or decline unsafe conditions (A = 0.833), and ST11, role clarity and ethical boundary management (*A* = 0.831). ST9, setting- and modality-specific professional competence (*A* = 0.767), and ST13, continuing professional development across the career span (*A* = 0.764), ranked fourth and fifth, respectively. ST10, trauma- and emotion-informed interpreting competence, satisfied the mean-threshold (*d* = 0.165) and defuzzification (*A* = 0.627) criteria, although its expert agreement of 60.00% fell below the required level. It was therefore excluded from the final framework.

Within D3, five of the seven candidate sub-themes were retained. ST14, peer consultation and mutual support, ranked first (*A* = 0.820), followed by ST17, collaborative briefing and case coordination (*A* = 0.784). ST20, access to qualified psychological and trauma-related care, ranked third (*A* = 0.691), while ST15, professional mentoring (*A* = 0.602), and ST19, formal post-assignment debriefing (*A* = 0.564), ranked fourth and fifth, respectively. ST16, inclusion as a member of the service team, and ST18, confidential professional supervision, each achieved 73.33% expert agreement, slightly below the required 75% level. Although both sub-themes satisfied the mean-threshold and defuzzification criteria, they were excluded from the final framework.

Within D4, 10 of the 11 candidate sub-themes were retained. ST21, manageable workload, scheduling, breaks, recovery time, and assignment rotation, received the highest priority within this dimension and the highest defuzzified value among all 31 candidate sub-themes (*A* = 0.902). ST29, safe reporting and effective responses to overload, adverse incidents, discrimination, and health concerns, ranked second (*A* = 0.804), followed by ST26, fair remuneration, including preparation and related professional time (*A* = 0.771). ST24, ergonomically and environmentally safe workspaces (*A* = 0.682), and ST25, access to reliable technology and technical support (*A* = 0.680), ranked fourth and fifth. The remaining retained sub-themes were ST27, employment security and fair contracting (*A* = 0.671); ST28, professional recognition, respect, and status (*A* = 0.640); ST31, organizational protection from abusive, threatening, or violent conduct (*A* = 0.638); ST23, vocal and musculoskeletal health protection (*A* = 0.513); and ST30, protected and remunerated access to organizational support resources (*A* = 0.500). ST22, acoustic and hearing safety, satisfied the mean-threshold (*d* = 0.155) and defuzzification (*A* = 0.562) criteria, while its expert agreement of 66.67% fell below the required level. It was therefore excluded.

Across the four dimensions, the highest-ranked sub-themes were emotional self-regulation during assignments in D1, assignment-specific contextual and terminological preparation in D2, peer consultation and mutual support in D3, and manageable workload, scheduling, breaks, recovery time, and assignment rotation in D4. The four exclusions resulted exclusively from expert-agreement percentages below 75%, since every candidate sub-theme satisfied the mean-threshold and defuzzification criteria. These findings indicate that the exclusions reflected insufficient convergence among individual expert judgments, even though the corresponding group-level threshold and importance values remained acceptable.

### 4.3 Final consensus framework

Following the FDM analysis, the final cross-context core framework comprised all four dimensions and 27 of the 31 candidate sub-themes, representing an overall retention rate of 87.10%. Although ST10, ST16, ST18, and ST22 satisfied the mean-threshold and defuzzification criteria, they fell below the required expert-agreement criterion, were not retained for the final framework, and should be viewed as context-specific support factors requiring further validation. Figure 2 presents the 27 retained sub-themes according to their dimensions and within-dimension priority rankings.

**Figure 2:**
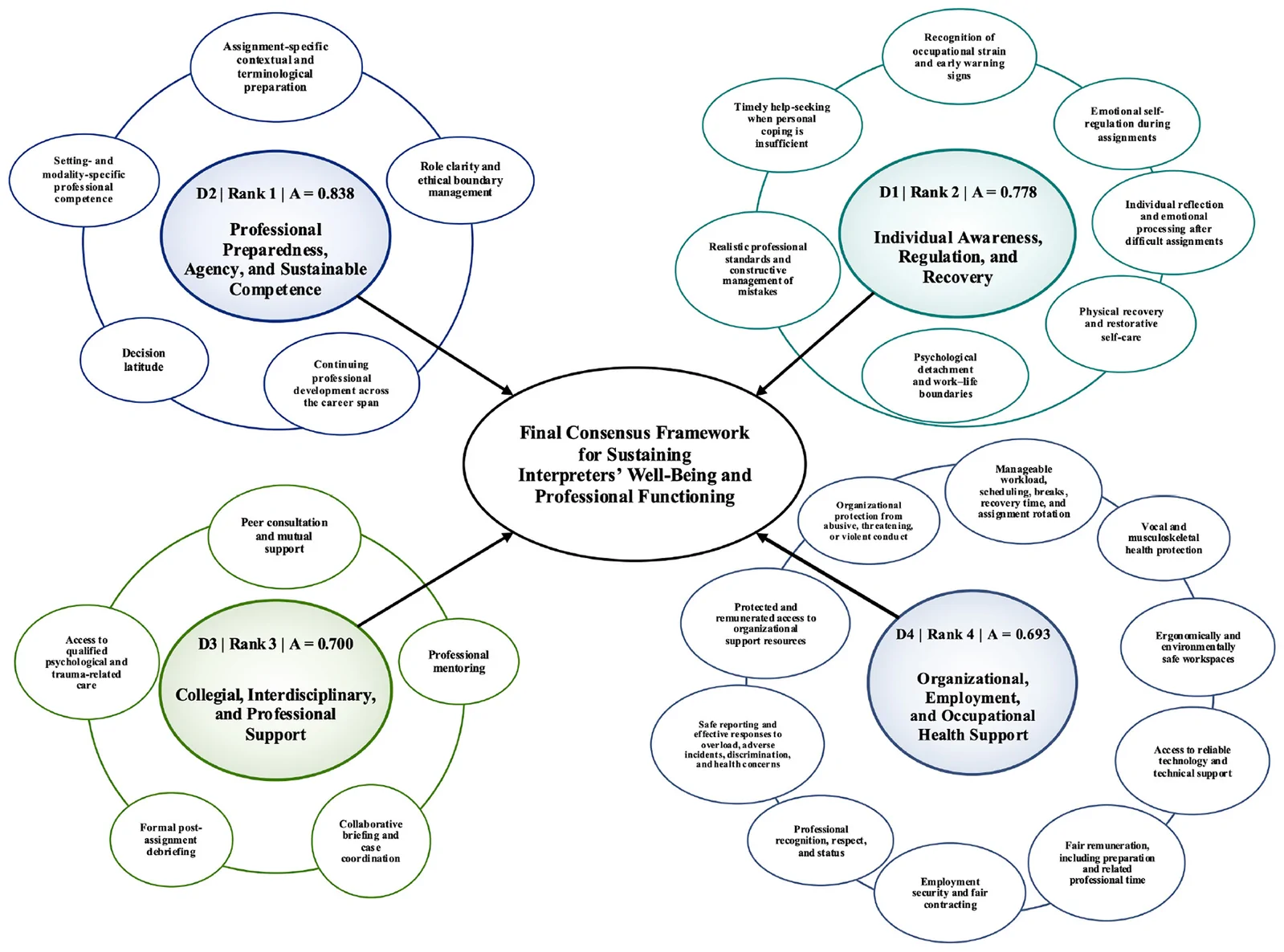
Final consensus framework for sustaining interpreters' wellbeing and professional functioning.

## 5 Discussion

This study developed and evaluated a framework for sustaining interpreters' wellbeing and professional functioning. The FDM retained all four proposed dimensions and 27 of the 31 candidate sub-themes, corresponding to a sub-theme retention rate of 87.10%. The resulting framework encompasses individual awareness, regulation, and recovery; professional preparedness, agency, and sustainable competence; collegial, interdisciplinary, and professional support; and organizational, employment, and occupational health support. It brings together resources that previous studies have frequently examined separately, including coping and recovery practices (1, 5), professional preparation and agency (4, 8, 29), collegial and interdisciplinary support (15, 30), and organizational and employment conditions (7, 31, 32). The four-dimensional structure is consistent with the job demands-resources model, which locates resources at different levels of the occupational environment (47). In the present study, the framework represents expert consensus on the perceived importance of these resources. Relationships among the dimensions and their associations with wellbeing outcomes were not examined.

At the dimension level, D2 received the strongest relative endorsement within the panel (*A* = 0.838), followed by D1 (*A* = 0.778), D3 (*A* = 0.700), and D4 (*A* = 0.693). These rankings represent the relative judgments of the 15 participating experts and therefore do not establish a universal hierarchy of occupational-health determinants. Nine panel members were primarily identified as interpreter educators or trainers, representing 60.0% of the panel. This composition may have increased the salience of preparation, competence, professional agency, and continuing development. In addition, the dimension rankings also provide no evidence that D2 has greater causal importance than workload, remuneration, employment security, occupational safety, or organizational protection. All four dimensions satisfied the predefined retention criteria. A lower dimension-level rank therefore indicates lower relative endorsement within this panel and carries no implication that the dimension is dispensable.

At the sub-theme level, the three highest relative importance ratings were recorded for ST21, manageable workload, scheduling, breaks, recovery time, and assignment rotation (*A* = 0.902); ST8, assignment-specific contextual and terminological preparation (*A* = 0.860); and ST12, decision latitude, including the ability to pause, seek assistance, or decline unsafe conditions (*A* = 0.833). The three lowest relative importance ratings were recorded for ST30, protected and remunerated access to organizational support resources (*A* = 0.500); ST23, vocal and musculoskeletal health protection (*A* = 0.513); and ST18, confidential professional supervision (*A* = 0.551). ST30 and ST23 met all three retention criteria, whereas ST18 fell below the required expert-agreement threshold. These values indicate relative endorsement within the present panel and should not be interpreted as population-level estimates of importance.

Within D1, all seven candidate sub-themes met the retention criteria. Emotional self-regulation during assignments received the strongest relative endorsement (*A* = 0.807), followed by recognition of occupational strain and early warning signs (*A* = 0.767) and individual reflection and emotional processing after difficult assignments (*A* = 0.744). Previous research has described attentional control, breathing, reflection, and awareness of emotional responses as strategies used when managing demanding assignments (1, 2, 8). In addition, psychological detachment has been identified as an important component of recovery from work-related demands during non-work time (48). In the present study, physical recovery and restorative self-care and psychological detachment and work-life boundaries both had a reported rounded defuzzified value of (*A* = 0.698), indicating comparable levels of endorsement after rounding. Realistic professional standards, constructive management of mistakes, and timely help-seeking were also retained. Their inclusion is consistent with previous findings associating maladaptive perfectionistic tendencies with emotional exhaustion among interpreters (27). Together, the D1 findings position individual support as a combination of awareness, in-assignment regulation, post-assignment recovery, appropriate professional standards, and timely recourse to further assistance. These resources form one part of the wider framework and should be considered alongside the professional, relational, and organizational conditions surrounding interpreting work (1, 5).

Within D2, assignment-specific contextual and terminological preparation received the strongest relative endorsement (*A* = 0.860), followed closely by professional decision latitude (*A* = 0.833) and role clarity and ethical boundary management (*A* = 0.831). Previous studies have described inadequate assignment information, unfamiliar terminology, unclear role expectations, and restricted professional agency as potential sources of uncertainty and occupational strain in interpreting work (1, 4, 8, 15, 29). The strong endorsement of these three factors suggests that the panel viewed preparation and professional agency as closely related aspects of sustainable practice. Preparation may help interpreters anticipate the linguistic and contextual demands of an assignment, while role clarity and decision latitude concern their ability to respond when assignment conditions change or become unsafe (8, 15, 29). Advance case information, terminology resources, briefing procedures, clear role protocols, and policies permitting interpreters to pause, seek assistance, request changes, or decline unsafe assignments are possible ways of operationalizing these factors. Their effectiveness would need to be evaluated in particular professional settings. Setting- and modality-specific competence and continuing professional development were also retained, extending D2 from immediate assignment readiness to the maintenance of professional competence across the career span (1, 4).

Within D3, peer consultation and mutual support received the strongest relative endorsement (*A* = 0.820), followed by collaborative briefing and case coordination (*A* = 0.784). Professional mentoring, formal post-assignment debriefing, and access to qualified psychological and trauma-related care also met the retention criteria. Relevant studies have described peer interaction as a source of practical advice, shared reflection, professional validation, and emotional support (5, 8, 15). Specifically, collaborative briefing has been recognized as a means of clarifying case background, assignment expectations, sensitive content, and team procedures (8, 15). The retained factors span several forms of assistance, from informal collegial exchange and assignment-specific coordination to structured reflection and specialist psychological care. However, their relative positions indicate the strength of endorsement within this panel and do not establish comparative effectiveness. Peer networks, mentoring arrangements, briefing procedures, debriefing pathways, and referral systems may be appropriate in different circumstances, depending on the professional setting, availability of qualified providers, confidentiality requirements, and continuity of the interpreting team (1, 15, 30).

Finally, within D4, ST21, which combined manageable workload, scheduling, breaks, recovery time, and assignment rotation, received the highest defuzzified value among all 31 candidates (*A* = 0.902), although D4 ranked fourth at the dimension level. This contrast reflects the different levels at which the experts made their judgments. Dimension ratings concerned broad areas of support, while sub-theme ratings concerned specific practices, resources, or working conditions. The lower aggregate ranking of D4 therefore does not diminish the strong endorsement of ST21 or the relevance of the other organizational factors retained within that dimension. Previous studies have associated work intensity, repeated exposure to demanding assignments, interpreting frequency, insufficient recovery, and dissatisfaction with employment conditions with psychological distress or emotional exhaustion among interpreters (1, 6–8, 31). Its position as the highest-rated sub-theme suggests that work design and recovery were particularly prominent item-level concerns for the panel. However, the present study did not evaluate whether modifying any individual component would improve interpreters' wellbeing.

Notably, the elements within ST21 were grouped as related work-design and recovery provisions that generally fall under organizational responsibility (47, 48). Its defuzzified value applies to the composite statement presented to the experts. The analysis cannot identify whether their endorsement was driven primarily by workload volume, scheduling arrangements or predictability, protected breaks, recovery time, assignment rotation, or the combined provision of these elements. Therefore, while the present indicates strong panel endorsement of the broader work-design issue, the relative importance of its individual components remains unresolved. In addition, the organizational dimension also encompassed fair remuneration, employment security and fair contracting, safe reporting, reliable technology, safe workspaces, professional recognition, protected access to organizational support resources, occupational-health protection, and protection from abusive or threatening conduct. Earlier research has described financial insecurity, inadequate professional recognition, limited workplace support, technological difficulties, and exposure to hostile conduct as relevant features of interpreters' working environments (5, 28, 30–32). The retention of these factors places responsibility for interpreter wellbeing beyond personal coping alone. Workload arrangements, compensation, employment conditions, technology, reporting procedures, and protection from harmful conduct are potential areas for organizational action. Thus, their feasibility and effects require evaluation within the employment structures and professional settings in which they would be implemented.

Regarding the four sub-themes that were not retained, expert agreement fell below the required 75% threshold for trauma- and emotion-informed interpreting competence (ST10, 60.00%), inclusion as a member of the service team (ST16, 73.33%), confidential professional supervision (ST18, 73.33%), and acoustic and hearing safety (ST22, 66.67%). All four nevertheless satisfied the mean-threshold and defuzzification criteria, indicating positive overall evaluations without sufficient convergence for inclusion in the proposed cross-context core. Their perceived relevance may vary across interpreting modalities, professional settings, employment arrangements, and forms of occupational exposure. For example, trauma-informed competence may receive greater emphasis in refugee, healthcare, legal, or humanitarian contexts (1, 15, 30). Team inclusion may be particularly relevant where interpreters work regularly within established interdisciplinary teams (15, 30). Supervision arrangements may differ across sectors and professional systems (1, 16), while acoustic safety may have greater relevance in audio-intensive spoken-language and remote interpreting (28). Therefore, future studies should evaluate these four sub-themes across interpreting modalities, professional settings, working language pairs, employment arrangements, and regions of practice to determine whether they warrant inclusion as context-specific components of the framework.

Theoretically, the framework elaborates the resource component of the job demands-resources model for interpreting work by distinguishing resources located at individual, professional, relational, and organizational levels (47). It also clarifies the distinction between support resources and possible outcomes. Professional meaning, compassion satisfaction, job satisfaction, and career continuity may reflect the consequences of sustainable working conditions, while the retained sub-themes describe practices, competencies, relationships, and organizational provisions that may contribute to those conditions (6, 13, 31). By placing individual coping resources alongside professional agency, collegial support, employment conditions, and occupational-health protection, the framework presents interpreter wellbeing as a shared concern involving both interpreters and the systems in which they work. However, the present study did not test pathways among demands, resources, and outcomes. The four dimensions therefore constitute a content framework whose explanatory and predictive relationships require further investigation.

Practically, the framework provides a structured basis for selecting support strategies for local development and evaluation. Interpreter education and training programs may use D1 and D2 to review their coverage of assignment preparation, emotional regulation, role clarity, decision latitude, professional boundaries, and help-seeking. Professional associations and interpreting-service providers may use D3 to examine peer consultation, mentoring, briefing, debriefing, and referral arrangements. Employers, agencies, platforms, and service institutions may use D4 to review workload design, recovery provisions, remuneration, contracting, technology, reporting procedures, and organizational protection. Nevertheless, the framework provides a general approach to supporting interpreters' wellbeing and professional functioning across contexts. Local priorities should therefore be established in consultation with interpreters and relevant stakeholders, as the relevance, feasibility, acceptability, accessibility, equity, cost, and effectiveness of particular support strategies may vary across settings.

Generalization of these findings requires caution. First, the initial framework was developed through an English-language targeted narrative review conducted primarily through Google Scholar. Supplementary searching expanded the evidence base, although relevant publications may have been missed. Second, the framework reflects the judgments of 15 purposively selected experts. Educators and trainers formed 60.0% of the panel, while four participants were identified primarily as practicing interpreters and one as a manager of interpreting services. A panel with a different professional composition may assign different priorities. Third, information on spoken-language or signed-language interpreting, primary interpreting setting, working language pairs, employment arrangement, country or region of practice, and years of interpreting practice separately from total relevant professional experience was unavailable. The extent to which the panel represented different areas of interpreting practice could therefore not be determined, and subgroup differences could not be examined. Fourth, while the questionnaire assessed perceived importance, it failed to assess feasibility, accessibility, cost, implementation responsibility, or effectiveness. The findings should consequently be interpreted as evidence of consensus and relative endorsement within this panel. In addition, the composite wording of several sub-themes creates a further limitation. In particular, the value of *A* = 0.902 for ST21 applies to the combined statement as it was presented to the experts. The analysis thus cannot determine whether the experts primarily endorsed manageable workload, scheduling or predictability, protected breaks, recovery time, assignment rotation, or their combined provision. These elements were grouped on the basis of their shared organizational location and their relevance to work design and recovery. Their grouping does not establish that they are equally important or operate in the same way. Similar composite sub-themes may also conceal differences in the perceived importance of their individual components. More generally, FDM establishes content consensus and relative priority, yet it could not provide evidence of predictive validity, causal relationships, or intervention effects.

Further research is needed to examine the stability and practical value of the framework across interpreting contexts. The consensus process could be repeated with larger panels that include broader representation of practicing interpreters, service managers, occupational-health professionals, and providers of psychological or supervisory support. Future studies should collect interpreting modality, professional setting, working languages, employment arrangement, region of practice, and years of interpreting practice prospectively. This information would permit clearer assessment of panel composition and comparisons across professional groups. The four context-sensitive candidates should also be reassessed in settings where their relevance is expected to be greater. Additionally, ST21 and other composite sub-themes should be separated into individual statements so that workload, scheduling, predictability, breaks, recovery time, and rotation can be evaluated independently. Subsequent scale-development studies may examine the structure and measurement properties of the retained framework. Longitudinal studies could investigate associations between access to the retained resources and changes in interpreters' wellbeing and professional functioning. Implementation and intervention studies would then be needed to determine the feasibility and effects of specific support strategies under defined working conditions. These refinements would provide stronger evidence regarding the framework's applicability across interpreting contexts and the suitability of different forms of support for specific settings and modalities.

## 6 Conclusion

To end, this study developed a consensus-based framework integrating individual, professional, relational, and organizational forms of support for interpreters' wellbeing and professional functioning. The final framework retained four dimensions and 27 sub-themes, with professional preparedness, agency, and sustainable competence receiving the highest dimension-level priority and manageable workload, scheduling, breaks, recovery time, and assignment rotation emerging as the highest-priority sub-theme overall. These findings indicate that the panel viewed individual resilience and self-care as only part of a broader support system encompassing adequate preparation, professional decision latitude, clear ethical boundaries, peer and interdisciplinary support, fair employment conditions, safe working environments, and organizational protection. The framework therefore provides a structured basis for interpreter education, professional development, organizational policy, and occupational-health planning, while allowing context-specific adaptation across interpreting settings and modalities. Given the exploratory nature of the FDM, the framework should thus be regarded as a foundation for subsequent measurement, validation, and intervention research aimed at determining how these support factors operate in practice and whether their implementation improves interpreters' psychological wellbeing, physical health, professional performance, and career sustainability.

## Data Availability

The datasets presented in this article are not readily available because they comprise de-identified expert questionnaire responses from a small panel, and combinations of demographic and professional characteristics may permit deductive identification of participants. Requests to access appropriately de-identified data should be directed to Yanfei Li at yanfei.li@taru.edu.cn and will be considered in accordance with the approved ethics procedures, participant-confidentiality requirements, and institutional policies.
