# Supplementary material for: Support factors for sustaining interpreters' wellbeing and professional functioning: a fuzzy Delphi study
Source: Front Public Health. 2026 Oct 6;14:1941746. doi: 10.3389/fpubh.2026.1941746 (PMC13680223; doi:10.3389/fpubh.2026.1941746)
Supplement: Supplementary file 1 [file Data_Sheet_1.docx]

**Contents of the Supplementary Material**

[***Table S1.*** Source documents contributing directly to the development or refinement of the framework.	1](#_Toc238431999)

[***Table S2.*** Conceptual and literature mapping of the 31 refined candidate sub-themes.	7](#_Toc238432000)

[***Table S3.*** Changes from the draft framework to the refined preliminary framework.	19](#_Toc238432001)

[***Table S4.*** Refined preliminary framework for sustaining interpreters’ well-being and professional functioning.	25](#_Toc238432002)

[***Table S5.*** FDM questionnaire used for expert evaluation: background-information items and seven-point importance-rating sections.	29](#_Toc238432003)

[**References**	32](#_Toc238432004)

# ***Table S1.*** Source documents contributing directly to the development or refinement of the framework.

| Source ID | Publication | Study or source type and context | Documented contribution to the framework | Stage of contribution |
| --- | --- | --- | --- | --- |
| S01 | [Alhawamdeh and Zhang (2021), *Wellness of interpreters: Stress-related occupational hazards and possible solutions*](https://doi.org/10.14456/nvts.2021.6) | Qualitative interviews with five interpreters from Jordan, China, Iraq, and the United States, with attention to working conditions during COVID-19 | Recognition of strain, emotional regulation, restorative self-care, help-seeking, setting-related preparedness, and access to psychological support: ST1, ST2, ST4, ST7, ST9, and ST20 | Draft development; retained during refinement |
| S02 | [Bakker and Demerouti (2017), *Job demands-resources theory: Taking stock and looking forward*](https://doi.org/10.1037/ocp0000056) | Theoretical review of the job demands-resources model | Supplied a resource-based occupational-health perspective for organizing support at individual, professional, relational, and organizational levels | Higher-order conceptual organization |
| S03 | [Brunale and Sannolla (2026), *Wellbeing promoting practices for interpreters*](https://doi.org/10.4324/9781003586647-15) | Scholarly synthesis of health- and well-being-promoting practices drawn from interpreting research, professional associations, and professional practice | Broad support for awareness, emotional regulation, self-care, detachment, help-seeking, trauma awareness, decision latitude, peer and mentoring support, briefing, debriefing, psychological care, workload management, hearing, voice, musculoskeletal health, and workspaces: ST1-ST7, ST10, ST12-ST15, ST17, and ST19-ST24 | Draft development and refinement |
| S04 | [Chen (2023), *The interplay between psychological well-being, stress, and burnout: Implications for translators and interpreters*](https://doi.org/10.1016/j.heliyon.2023.e18589) | Scholarly review of psychological well-being, stress, and burnout among translators and interpreters | Supported career-long professional development, psychological resources, and the initial distinction between support factors and possible outcomes: ST13 and the original outcome-oriented ST31 | Draft development; outcome classification during refinement |
| S05 | [Courtney and Phelan (2019), *Translators’ experiences of occupational stress and job satisfaction*](https://doi.org/10.12807/ti.111201.2019.a06) | Cross-sectional survey of 474 translators in the United Kingdom and Ireland | Work pace, long hours, workload variation, technological change, remuneration, employment uncertainty, agency treatment, and professional recognition: ST21 and ST25-ST28 | Draft development and organizational refinement |
| S06 | [Crezee and Lai (2022), *Interpreters’ resilience and self-care during pandemic restrictions in Australia and New Zealand*](https://doi.org/10.14456/nvts.2022.23) | Qualitative study of community interpreters working remotely and in person during the pandemic | Awareness, emotional regulation, physical self-care, detachment, help-seeking, professional development, peer support, mentoring, workload and breaks, acoustic conditions, workspace, technology, employment conditions, and access to support: ST1-ST5, ST7, ST13-ST15, ST21-ST22, ST24-ST25, ST27, and ST30 | Draft development and refinement |
| S07 | [Dean et al. (2025), *Video Relay Service interpreters’ experiences with caller behavior: An occupational health risk call to action*](https://doi.org/10.3390/healthcare13172116) | Mixed-methods survey of 345 American Video Relay Service interpreters | Technology-mediated working conditions, reporting of harmful incidents, limited organizational responses, and protection from abusive, sexual, racial, threatening, or illegal caller conduct: ST25, ST29, and ST31 | Refinement, particularly the addition of ST31 and expansion of ST29 |
| S08 | [Geiling, Böttche, et al. (2022), *A comparison of interpreters’ wellbeing and work-related characteristics in the care of refugees across different work settings*](https://doi.org/10.1186/s12889-022-14034-7) | Nationwide German survey with 158 interpreters at the first time point and 63 at the second, covering psychotherapy, counseling, medical, and public-authority settings | Setting-specific demands, work-related characteristics, workload and exposure, and the treatment of compassion satisfaction as a possible outcome: ST9, ST21, and the original outcome-oriented ST31 | Draft development and outcome classification |
| S09 | [Geiling et al. (2021), *Mental health and work experiences of interpreters in the mental health care of refugees: A systematic review*](https://doi.org/10.3389/fpsyt.2021.710789) | Systematic review of 25 studies concerning spoken-language interpreters in refugee mental-health care | Broad evidence concerning emotional strain, coping, preparation, role ambiguity, peer support, briefing, supervision, debriefing, working conditions, payment, recognition, and organizational support: ST1, ST3, ST5, ST7-ST12, ST14, ST17-ST21, and ST26-ST30 | Draft development and refinement |
| S10 | [Geiling, Knaevelsrud, et al. (2022), *Psychological distress, exhaustion, and work-related correlates among interpreters working in refugee care*](https://doi.org/10.1080/20008198.2022.2046954) | Nationwide German cross-sectional survey of 164 interpreters working in refugee care | Supervision, traumatic exposure, workload, payment dissatisfaction, professional recognition, and broader work-related support: ST18, ST21, ST26-ST28, and ST30 | Draft development and refinement |
| S11 | [Goldhirsch et al. (2022), *Addressing vicarious trauma and emotional stress among medical interpreters through care, partnership and respect*](https://doi.org/10.14456/nvts.2022.24) | Practice-based account of a clinician-interpreter support and education initiative in palliative care | Assignment preparation, emotion-informed competence, role clarification, peer exchange, team inclusion, briefing, supervision-related reflection, debriefing, psychological support, professional respect, and organizational access to support: ST8, ST10-ST11, ST14, ST16-ST20, and ST28-ST30 | Draft development and refinement |
| S12 | [Graves et al. (2023), *Compassion and trauma: Occupational health experiences of certified medical interpreters in five U.S. states*](https://doi.org/10.1177/21650799231167586) | Structured online survey of 199 certified medical interpreters in five U.S. states; the qualitative thematic analysis examined responses from the 55 participants who completed the open-ended question | Professional role, trauma-related competence, emotional distancing, collegial and workplace support, team inclusion, interpreter safety, and professional recognition: ST10-ST11, ST14, ST16-ST17, ST20, ST28, and ST31 | Refinement of professional, relational, and organizational factors |
| S13 | [Hanft-Robert and Mösko (2024), *Community interpreting in Germany: Results of a nationwide cross-sectional study among interpreters*](https://doi.org/10.1186/s12889-024-18988-8) | Nationwide cross-sectional survey of 873 community interpreters in Germany | Preparation information, competence and training, access to supervision, interpreting frequency, payment, employment security, professional recognition, and access to support: ST8-ST9, ST13, ST18, ST21, ST26-ST28, and ST30 | Refinement of professional, employment, and organizational factors |
| S14 | [Hapfelmeier et al. (2025), *Secondary traumatic stress in interpreters for refugees: Why training and supervision matter*](https://doi.org/10.3390/psychiatryint6030091) | Cross-sectional survey of 64 interpreters working with refugees in Austria, Germany, and Switzerland | Recognition of secondary-trauma symptoms, help-seeking, trauma-informed training, peer support, supervision, debriefing, and protected access to support: ST1, ST7, ST10, ST14, ST18-ST19, and ST30 | Draft development and refinement |
| S15 | [Korpal and Mellinger (2022), *Self-care strategies of professional community interpreters: An interview-based study*](https://doi.org/10.1075/tcb.00069.kor) | Semi-structured interviews with 20 professional community interpreters; analysis across awareness, balance, flexibility, physical health, social support, and spirituality | Awareness, emotional regulation, physical self-care, psychological detachment, help-seeking, and peer or social support: ST1-ST5, ST7, and ST14 | Draft development |
| S16 | [Leanza et al. (2025), *Interpreting is an emotional effort: Public service interpreters’ coping strategies in emotionally charged situations*](https://doi.org/10.1177/27523810241310909) | Qualitative narrative study of 21 signed- and spoken-language public-service interpreters | In-assignment emotional regulation, individual reflection, role and boundary management, collaborative responses, team inclusion, and assignment coordination: ST2-ST3, ST11, and ST16-ST17 | Draft development and relational refinement |
| S17 | [Lim et al. (2022), *“We need a little help”: A qualitative study on distress and coping among pediatric medical interpreters*](https://doi.org/10.21037/jhmhp-22-23) | Qualitative interviews with 13 Spanish-English pediatric medical interpreters in a U.S. hospital | Emotional regulation, reflection, recovery, detachment, help-seeking, preparation, trauma competence, role and agency, peer and mentoring support, team inclusion, debriefing, psychological care, workload, and organizational access: ST2-ST5, ST7-ST12, ST14-ST16, ST19-ST21, and ST30 | Draft development and refinement |
| S18 | [Louzán et al. (2025), *Ergonomic and psychosocial risks in sign language interpreters and guide-interpreters: A systematic review and bibliometric analysis*](https://doi.org/10.1080/00140139.2025.2519871) | Systematic review of 33 studies and bibliometric analysis concerning sign-language interpreters and guide-interpreters | Repetitive movement, sustained posture, musculoskeletal risk, workplace conditions, workplace violence, reporting, and organizational protection: ST23-ST24 and ST29-ST31 | Refinement of occupational-health and organizational-protection factors |
| S19 | [Mack and Amato (Eds.) (2026), *Interpreters’ health and wellbeing: An overview across modes, modalities and settings*](https://doi.org/10.4324/9781003586647) | Edited multidisciplinary scholarly volume covering spoken- and signed-language interpreting, hearing, voice, musculoskeletal health, cognitive and emotional demands, technology, and physical work environments | Setting- and modality-specific competence, acoustic and hearing safety, vocal and musculoskeletal protection, safe workspaces, technology-related demands, professional conditions, and organizational protection: ST9 and ST22-ST25, ST27-ST28, and ST31 | Draft development and occupational-health refinement |
| S20 | [Palmer et al. (2025), *Burnout among sign language interpreters working in postsecondary institutions*](https://doi.org/10.1075/intp.00122.pal) | National U.S. cross-sectional survey of sign-language interpreters working in postsecondary institutions | Burnout, interpreter attrition, health-related support needs, and the distinction between organizational support factors and career continuity as a possible outcome: contextual support for ST29 and the original outcome-oriented ST31 | Draft development and outcome classification |
| S21 | [Polat Ulaş (2022), *Emotional challenges of interpreters working with refugees in Türkiye*](https://doi.org/10.14456/nvts.2022.22) | Qualitative interviews with 29 interpreters working with refugees in nongovernmental, legal, and healthcare settings | Help-seeking, trauma-informed competence, role and ethical boundaries, debriefing and psychological care, discrimination, and organizational protection: ST7, ST10-ST11, ST19-ST20, and ST29-ST31 | Draft development and organizational refinement |
| S22 | [Schwenke (2012), *Sign language interpreters and burnout*](https://digitalcommons.unf.edu/joi/vol20/iss1/7/) | Cross-sectional survey of 117 sign-language interpreters examining burnout, social support, and decision latitude | Professional decision latitude, collegial support, supervision, and the workload component of work design: ST12, ST14, ST18, and ST21 | Draft development |
| S23 | [Schwenke et al. (2014), *Sign language interpreters and burnout: The effects of perfectionism, perceived stress, and coping resources*](https://doi.org/10.1075/intp.16.2.04sch) | Quantitative study of perfectionism, perceived stress, coping resources, and burnout among sign-language interpreters | Realistic professional standards and constructive management of mistakes: ST6 | Draft development |
| S24 | [Sonnentag and Fritz (2007), *The Recovery Experience Questionnaire: Development and validation of a measure for assessing recuperation and unwinding from work*](https://doi.org/10.1037/1076-8998.12.3.204) | General occupational-health scale-development and validation study | Conceptual definition of psychological detachment and recovery during non-work time: ST5 | Conceptual refinement of ST5 |
| S25 | [Valero-Garcés (2015), *The impact of emotional and psychological factors on public service interpreters: Preliminary studies*](https://doi.org/10.12807/ti.107203.2015.a07) | Review of preliminary interview- and questionnaire-based studies involving public-service, conference, mental-health, and trainee interpreters | Assignment preparation, setting competence, role-related pressures, formal debriefing, and psychological support: ST8-ST9, ST11, and ST19-ST20 | Draft development |
| S26 | [Yan and Jia (2025), *Occupational stress and turnover intentions among simultaneous interpreters: The mediating role of burnout and the moderating role of grit*](https://doi.org/10.1080/0907676X.2025.2478403) | Quantitative survey and structural equation modeling involving 478 simultaneous interpreters in China | Career-long professional development, supportive working environments, workload and occupational stress, professional sustainability, and classification of turnover or career continuity as an outcome: ST13, ST21, and ST28; original outcome-oriented ST31 | Draft development and outcome classification |

***Note.*** Source IDs are used in Tables S2–S4. Each journal article, review, scholarly chapter, or edited volume was counted once. The Mack and Amato edited volume and the Brunale and Sannolla chapter were counted separately since distinct content was extracted from the wider volume and the separately authored Chapter 11. The “Documented contribution” column covers both draft development and refinement; references to the original outcome-oriented ST31 denote the candidate removed during content and face review. Primary studies summarized within reviews were counted only when they were independently reviewed and listed in Table S1. Publications used solely to justify Delphi procedures, FDM calculations, expert-panel selection, instrument validation, analytical thresholds, defuzzification, or ranking rules were excluded from this corpus. Table S2 provides the detailed item-level mapping for the refined candidate set.

# ***Table S2.*** Conceptual and literature mapping of the 31 refined candidate sub-themes.

| Dimension and candidate sub-theme | Definition and conceptual boundary | Ecological level, provider, and proposed protective mechanism | Illustrative operationalization and relevant contexts/modalities | Supporting source IDs |
| --- | --- | --- | --- | --- |
| D1. Individual awareness, regulation, and recovery | | | | |
| D1, ST1. Recognition of occupational strain and early warning signs | Awareness of emerging cognitive, emotional, behavioral, and physical signs of work-related strain. This factor concerns recognition and monitoring, distinct from in-assignment regulation under ST2 and external assistance under ST7. | Individual level; interpreter-led. Early recognition may enable timely recovery, adjustment of work demands, or help-seeking before strain becomes prolonged. | Self-monitoring checklists; reflective review of fatigue, intrusive recollections, sleep disruption, emotional numbing, reduced concentration, cynicism, or declining effectiveness. Relevant across modalities and settings, especially during frequent, prolonged, or emotionally demanding work. | S01, S03, S06, S09, S14, S15 |
| D1, ST2. Emotional self-regulation during assignments | Strategies used during an assignment to regulate arousal, attention, and emotional reactions while maintaining communicative functioning. This factor is temporally distinct from post-assignment reflection under ST3. | Individual level; interpreter-led. Regulation strategies may help maintain attentional control and functional engagement during emotionally or cognitively demanding encounters. | Grounding, controlled breathing, mindful awareness, attentional refocusing, brief emotional distancing, and concentration on the interpreting task. Applicable across modalities, with particular relevance to simultaneous, healthcare, refugee, legal, crisis, and other high-intensity interpreting. | S01, S03, S06, S16, S17 |
| D1, ST3. Individual reflection and emotional processing after difficult assignments | Self-directed post-assignment processing through which the interpreter reviews and integrates emotional responses. This factor is distinct from facilitated debriefing under ST19 and recurring supervision under ST18. | Individual level; interpreter-led. Reflective processing may support emotional awareness, meaning-making, identification of unresolved concerns, and recognition of any need for further assistance. | Reflective writing, private case reflection, journaling, structured self-reflection, or confidential personal processing. Potentially relevant across modalities, with greater salience in healthcare, refugee, legal, humanitarian, mental-health, and other emotionally demanding settings. | S03, S09, S16, S17 |
| D1, ST4. Physical recovery and restorative self-care | Personal activities that replenish physical and psychological resources following work. This factor concerns restorative practices, distinct from cognitive disengagement under ST5 and organizationally provided recovery time under ST21. | Individual level; interpreter-led. Restorative activities may replenish depleted physical and psychological resources and support recovery between assignments. | Sleep, rest, exercise, hydration, relaxation, hobbies, movement, stretching, and other restorative routines. Relevant across modalities, particularly following prolonged, intensive, irregularly scheduled, or physically demanding interpreting. | S01, S03, S06, S15, S17 |
| D1, ST5. Psychological detachment and work-life boundaries | Mental disengagement from work-related thoughts during non-work time and maintenance of boundaries between professional and personal life. This factor is distinct from general restorative activities under ST4. | Individual level; interpreter-led, with organizational conditions influencing feasibility. Psychological detachment may limit continued work-related activation and preserve time and attention for recovery outside work. | Protected non-work time, transition routines, separation of home and work spaces, limits on work-related communication, and deliberate disengagement from cases. Relevant across settings, with particular salience for freelance, on-call, remote, and home-based interpreters. | S03, S06, S09, S15, S17, S24 |
| D1, ST6. Realistic professional standards and constructive management of mistakes | Maintenance of demanding yet attainable professional standards, accompanied by proportionate self-evaluation and constructive responses to errors. This factor is distinct from setting- and modality-specific competence under ST9. | Individual and professional-practice level; interpreter-led, with feedback from educators, mentors, peers, or supervisors. Constructive appraisal may reduce persistent self-criticism and enable learning from mistakes while maintaining professional accountability. | Realistic performance expectations, reflective error review, constructive feedback, acknowledgement of human limitations, and learning-oriented responses to mistakes. Relevant across modalities, especially in high-stakes or strongly performance-oriented settings. | S03, S23 |
| D1, ST7. Timely help-seeking when personal coping is insufficient | Recognition that personal coping resources have reached their limits, followed by timely access to collegial, supervisory, occupational-health, or psychological assistance. This factor concerns the interpreter’s help-seeking action, distinct from the availability of qualified care under ST20. | Individual-to-support interface; initiated by the interpreter, with peers, supervisors, managers, occupational-health personnel, or mental-health professionals providing assistance. Timely help-seeking may reduce prolonged unsupported strain and connect the interpreter with an appropriate level of support. | Contacting a trusted peer, mentor, supervisor, manager, occupational-health service, counselor, or trauma specialist. Relevant across modalities, especially when distress persists, personal functioning declines, or an assignment involves traumatic or highly distressing material. | S01, S03, S06, S09, S14, S15, S17, S21 |
| D2. Professional preparedness, agency, and sustainable competence | | | | |
| D2, ST8. Assignment-specific contextual and terminological preparation | Preparation for the content, terminology, participants, purpose, risks, and interactional demands of a particular assignment. This factor is assignment-specific, distinct from the broader professional competence represented by ST9. | Professional-practice level; undertaken by the interpreter and enabled by clients, coordinators, agencies, or service institutions. Adequate preparation may reduce avoidable uncertainty and improve readiness for linguistic, interactional, and emotional demands. | Advance case information, agendas, scripts, background documents, terminology resources, pre-assignment questions, and protected preparation time. Relevant across spoken- and signed-language interpreting, especially in conference, healthcare, legal, refugee, public-service, and specialist assignments. | S09, S11, S12, S13, S17, S25 |
| D2, ST9. Setting- and modality-specific professional competence | Knowledge and skills required for competent practice within a particular interpreting setting and modality, including relevant interactional, ethical, linguistic, and technological conventions. | Professional-practice level; developed by interpreters, educational institutions, training providers, professional associations, and employers. Alignment between competence and assignment demands may reduce uncertainty and support confident, safe, and context-appropriate practice. | Competence for healthcare, legal, refugee, conference, community, signed-language, simultaneous, telephone, video-mediated, or remote interpreting. Applicable across settings, with content tailored to the demands of each modality and professional context. | S01, S08, S09, S11, S13, S17, S19, S25 |
| D2, ST10. Trauma- and emotion-informed interpreting competence | Professional knowledge and skills for anticipating, recognizing, and managing emotionally demanding or trauma-related interpreting situations. This factor is distinct from personal emotional regulation under ST2 and therapeutic care under ST20. | Professional-practice level; developed by interpreters, educators, trainers, supervisors, and service institutions. Trauma- and emotion-informed competence may improve preparedness for emotionally intense encounters and facilitate appropriate boundaries, responses, and referrals. | Education concerning vicarious trauma, secondary traumatic stress, emotional dynamics, trauma-sensitive communication, warning signs, coping resources, and referral pathways. Especially relevant to refugee, asylum, healthcare, mental-health, legal, humanitarian, crisis, and violence-related interpreting. | S03, S09, S11, S12, S14, S17, S21 |
| D2, ST11. Role clarity and ethical boundary management | Understanding of the interpreter’s responsibilities and active management of confidentiality, neutrality, advocacy, cultural mediation, emotional involvement, and related ethical boundaries. This factor is distinct from the authority to influence assignment conditions under ST12. | Professional and institutional level; shared by interpreters, professional associations, service institutions, clients, and interdisciplinary teams. Clear roles and boundaries may reduce role conflict, competing expectations, and inappropriate responsibility. | Role protocols, ethical guidance, confidentiality procedures, explicit responsibility agreements, informed team discussions, and strategies for negotiating boundary conflicts. Relevant across modalities, especially in healthcare, legal, refugee, community, and other public-service settings. | S09, S11, S12, S16, S17, S21, S25 |
| D2, ST12. Decision latitude, including the ability to pause, seek assistance, or decline unsafe conditions | Professional discretion to influence how an assignment proceeds when communication quality, ethics, personal safety, or service-user safety is at risk. This factor concerns authority to act, distinct from knowledge of professional responsibilities under ST11. | Professional and organizational interface; exercised by the interpreter and enabled through organizational policies, contractual arrangements, and team practices. Greater decision latitude may allow interpreters to manage excessive or unsafe demands and align practice with professional and ethical requirements. | Requesting clarification, pausing an encounter, seeking assistance, requesting a colleague, negotiating assignment conditions, requesting reassignment, or declining unsafe work. Relevant across settings, especially during high-risk, poorly specified, excessively demanding, or ethically complex assignments. | S03, S09, S17, S22 |
| D2, ST13. Continuing professional development across the career span | Ongoing maintenance and extension of professional, setting-specific, technological, ethical, and well-being-related competence throughout an interpreter’s career. | Professional and institutional level; shared by interpreters, educational institutions, professional associations, employers, and service providers. Continuing development may sustain competence, facilitate adaptation to changing demands, and support career-long professional functioning. | Refresher education, specialist courses, modality training, technology training, coping education, mentoring-linked development, and paid professional-learning opportunities. Relevant across career stages, particularly during transitions into specialist settings or new technologies and modalities. | S03, S04, S06, S13, S19, S26 |
| D3. Collegial, interdisciplinary, and professional support | | | | |
| D3, ST14. Peer consultation and mutual support | Reciprocal exchange among interpreters of broadly comparable professional status for practical advice, emotional validation, shared learning, and temporary assistance. This factor is distinct from senior-led mentoring under ST15 and qualified supervision under ST18. | Collegial level; provided reciprocally by interpreter peers. Peer exchange may facilitate shared problem-solving, professional validation, normalization of occupational experiences, and reduced professional isolation. | Peer networks, buddy systems, informal check-ins, confidential case consultation, terminology exchange, mutual coverage, and moderated peer groups. Relevant across modalities, with particular value for freelance, geographically dispersed, remote, or professionally isolated interpreters. | S03, S06, S09, S11, S14, S15, S17, S22 |
| D3, ST15. Professional mentoring | Developmental guidance provided over time by a more experienced interpreter, particularly during entry into the profession, transition between settings, or exposure to increasingly complex work. | Collegial and professional-development level; provided by an experienced interpreter or formally appointed mentor. Mentoring may support gradual competence development, professional confidence, boundary formation, and adaptation to challenging assignments. | Named mentors, structured mentoring programs, observation, developmental feedback, career guidance, and staged exposure to demanding assignments. Especially relevant to early-career interpreters, newly qualified practitioners, and interpreters entering specialist settings or modalities. | S03, S06, S17 |
| D3, ST16. Inclusion as a member of the service team | Ongoing recognition and inclusion of interpreters within the professional team surrounding service delivery. This factor is distinct from assignment-specific briefing and coordination under ST17. | Interdisciplinary and organizational level; provided by service teams, managers, and institutions. Team inclusion may improve access to relevant information, professional recognition, coordination, and social integration. | Participation in relevant team communication, access to appropriate case information, acknowledgement of interpreting expertise, inclusion in team support arrangements, and consultation on interpreter-related decisions. Most applicable where interpreters work regularly with established teams, including healthcare, mental-health, refugee, social-service, and multidisciplinary legal settings. | S11, S12, S16, S17 |
| D3, ST17. Collaborative briefing and case coordination | Assignment-specific communication and planning among interpreters and other professionals before or during an encounter. This factor is distinct from continuing team membership under ST16 and post-assignment processing under ST19. | Interdisciplinary level; jointly provided by interpreters, case professionals, coordinators, and service teams. Briefing and coordination may clarify expectations, improve preparedness, and support a shared approach to sensitive or complex encounters. | Pre-encounter huddles, discussion of sensitive content, clarification of objectives and roles, agreement on interactional procedures, terminology review, and real-time case coordination. Especially relevant to healthcare, mental-health, refugee, legal, humanitarian, and other team-based assignments. | S03, S09, S11, S12, S16 |
| D3, ST18. Confidential professional supervision | Recurring, confidential, professionally focused reflection facilitated by a qualified supervisor. It addresses practice, emotional responses, ethical tensions, and role difficulties over time, distinct from case-specific debriefing under ST19 and therapeutic care under ST20. | Formal-support level; provided by a qualified professional supervisor with relevant interpreting or sector knowledge. Supervision may support reflective practice, integration of professional and emotional experiences, ethical decision-making, and timely identification of further support needs. | Scheduled individual or group supervision, qualified supervisors, confidentiality protocols, reflective case discussion, and employer-funded attendance. Potentially relevant where interpreters experience repeated emotional exposure or complex ethical demands, including healthcare, mental-health, refugee, legal, and humanitarian settings. | S03, S09, S10, S13, S14, S22 |
| D3, ST19. Formal post-assignment debriefing | Structured, case-specific processing facilitated by another person following a demanding assignment. This factor is distinct from self-directed reflection under ST3 and recurring professional supervision under ST18. | Relational and formal-support level; facilitated by a trained colleague, supervisor, coordinator, or team professional. Debriefing may support case review, emotional processing, identification of follow-up needs, and a sense of closure. | Facilitated post-case review, discussion of difficult events, review of professional responses, emotional processing, clarification of follow-up needs, and closure procedures. Especially relevant after demanding healthcare, refugee, legal, humanitarian, crisis, violence-related, or end-of-life assignments. | S03, S09, S11, S14, S17, S21, S25 |
| D3, ST20. Access to qualified psychological and trauma-related care | Availability of appropriately qualified mental-health support for distress requiring individualized or clinical expertise. This factor is distinct from professional supervision and case debriefing in provider qualification, purpose, and level of care. | Specialist formal-support level; provided by qualified counselors, psychologists, psychotherapists, psychiatrists, trauma specialists, or occupational-health professionals. Access may provide a pathway for confidential assessment, individualized support, treatment, and recovery. | Counseling, psychotherapy, trauma-focused care, employee-assistance referrals, bereavement support, crisis services, and confidential mental-health pathways. Relevant across modalities, with particular salience following trauma exposure, persistent distress, impaired functioning, or repeated work with highly distressing material. | S01, S03, S09, S11, S12, S17, S21, S25 |
| D4. Organizational, employment, and occupational health support | | | | |
| D4, ST21. Manageable workload, scheduling, breaks, recovery time, and assignment rotation | Organizational work design regulating the intensity, duration, sequence, predictability, and recovery demands of interpreting work. This candidate is composite, and its constituent elements require separate evaluation in subsequent research. | Organizational and job-design level; provided by employers, agencies, platforms, coordinators, and service institutions. Work-design provisions may improve the balance between occupational demands and recovery opportunities and limit cumulative exposure to intensive assignments. | Workload limits, predictable scheduling, protected breaks, recovery time following difficult cases, avoidance of consecutive high-intensity assignments, team interpreting, and assignment rotation. Relevant across modalities, especially simultaneous, conference, healthcare, refugee, crisis, remote, video-mediated, and high-volume interpreting. | S03, S05, S06, S08, S09, S10, S13, S17, S22, S26 |
| D4, ST22. Acoustic and hearing safety | Prevention of harmful sound exposure and excessive listening effort through safe equipment, sound levels, work procedures, and hearing-health monitoring. This factor is distinct from general workspace design under ST24 and technological reliability under ST25. | Organizational and occupational-health level; shared by employers, agencies, platform providers, technical personnel, occupational-health services, and interpreters. Acoustic controls may reduce hazardous sound exposure, excessive listening effort, and delayed responses to auditory symptoms. | Safe sound levels, suitable headsets, reliable audio transmission, acoustic-shock procedures, hearing protection, exposure monitoring, and assessment of persistent auditory symptoms. Most relevant to spoken-language, conference, simultaneous, telephone, remote, and video-mediated interpreting. | S03, S06, S19 |
| D4, ST23. Vocal and musculoskeletal health protection | Prevention of vocal strain and upper-body or repetitive-strain conditions associated with spoken- and signed-language interpreting. The candidate combines two bodily health areas within the occupational-health dimension. | Individual and organizational occupational-health level; shared by interpreters, employers, service providers, educators, ergonomics specialists, and occupational-health professionals. Preventive measures may reduce cumulative vocal or biomechanical load and encourage early responses to symptoms. | Vocal hygiene, hydration, microphone technique, vocal rest, posture variation, stretching, movement, task rotation, ergonomic education, and early assessment of pain or strain. Vocal provisions are especially relevant to spoken-language interpreting; musculoskeletal provisions have particular relevance to signed-language interpreting and prolonged seated or repetitive work. | S03, S18, S19 |
| D4, ST24. Ergonomically and environmentally safe workspaces | Physical work environments that support safe posture, visibility, audibility, concentration, movement, and physiological comfort. This factor is distinct from specific acoustic controls under ST22 and technological reliability under ST25. | Organizational and environmental level; provided by employers, agencies, institutions, venue operators, and platform providers, with interpreters contributing to home-workstation configuration. Suitable environments may reduce physical strain, sensory discomfort, fatigue, and avoidable interference with task performance. | Appropriate chairs and desks, screen positioning, booth dimensions, legroom, lighting, ventilation, temperature, visibility, movement space, and safe home workstations. Relevant to conference booths, institutional workplaces, signed-language settings requiring clear visibility, and remote or home-based interpreting. | S03, S06, S18, S19 |
| D4, ST25. Access to reliable technology and technical support | Availability of functional interpreting technologies and timely assistance when equipment, software, platforms, connectivity, or audio-visual systems fail. | Organizational and technical-infrastructure level; provided by employers, agencies, platform operators, service institutions, and technical-support teams. Reliable systems and timely assistance may reduce avoidable disruption, cognitive load, uncertainty, and prolonged exposure to technically compromised assignments. | Tested equipment, stable platforms, adequate connectivity, technical orientation, accessible troubleshooting, replacement equipment, pre-session checks, and incident support. Particularly relevant to remote, telephone, video-mediated, platform-based, and hybrid interpreting, with increasing relevance to technology-supported on-site work. | S05, S06, S07, S17, S19 |
| D4, ST26. Fair remuneration, including preparation and related professional time | Compensation that reflects both interpreting and the associated professional activities required to perform an assignment. This factor concerns payment adequacy and coverage, distinct from employment and contractual stability under ST27. | Employment and commissioning level; determined by employers, agencies, platforms, service purchasers, and contracting institutions. Fair remuneration may acknowledge the full scope of professional labor and reduce uncompensated work and financial strain. | Payment for interpreting, preparation, briefing, waiting, travel, documentation, debriefing, specialist competence, and cancelled assignments. Relevant to staff, freelance, agency, platform, community, conference, healthcare, legal, and other contractual arrangements. | S05, S09, S10, S13, S19 |
| D4, ST27. Employment security and fair contracting | Stable, transparent, and equitable employment or contractual arrangements for staff, freelance, agency, platform, and other interpreters. This factor concerns continuity and procedural fairness, distinct from the level and coverage of remuneration under ST26. | Employment and organizational level; provided by employers, agencies, platforms, commissioners, and service institutions. Security, transparency, and fair procedures may reduce employment uncertainty and support predictable access to work and occupational protections. | Clear contracts, predictable terms, job continuity, cancellation provisions, timely payment, transparent allocation of work, access to leave or insurance where applicable, and equitable treatment across employment categories. Especially relevant to freelance, temporary, agency, and platform-based interpreting. | S05, S06, S09, S10, S13, S19 |
| D4, ST28. Professional recognition, respect, and status | Organizational and professional acknowledgement of interpreting as skilled work requiring expertise, preparation, autonomy, and appropriate working conditions. This factor is distinct from assignment-level inclusion within a particular service team under ST16. | Organizational and professional-system level; provided by managers, institutions, service users, professional bodies, commissioners, and interdisciplinary colleagues. Recognition may strengthen professional legitimacy, respectful interaction, occupational identity, and interpreters’ ability to voice work-related concerns. | Respectful treatment, recognition in organizational policies, acknowledgement of specialist competence, consultation on interpreter-related decisions, appropriate titles, professional visibility, and inclusion in workforce planning. Relevant across interpreting modalities and employment settings. | S05, S09, S10, S11, S12, S13, S19 |
| D4, ST29. Safe reporting and effective responses to overload, adverse incidents, discrimination, and health concerns | Confidential and non-retaliatory channels through which interpreters can report occupational concerns, accompanied by timely review, feedback, and corrective action. This factor concerns reporting and organizational response, distinct from the specific protective measures under ST31. | Organizational governance and psychological-safety level; provided by employers, agencies, platforms, managers, service institutions, and designated safeguarding or occupational-health personnel. Safe reporting may enable earlier identification of hazards, institutional accountability, and corrective responses. | Confidential reporting channels, escalation pathways, incident documentation, anti-retaliation safeguards, managerial review, feedback to the reporter, risk assessment, and corrective action. Relevant across contexts, especially client-facing, remote, high-volume, healthcare, legal, public-service, and other settings involving elevated exposure to adverse incidents. | S07, S11, S12, S13, S18, S20, S21 |
| D4, ST30. Protected and remunerated access to organizational support resources | Formal access to training, supervision, debriefing, peer support, and psychological care during protected and paid professional time. This factor concerns the conditions governing access; the individual forms of support are specified elsewhere in the framework. | Organizational and employment level; provided by employers, agencies, platforms, service institutions, and professional organizations. Protected and remunerated access may reduce financial, scheduling, and employment-related barriers to the use of support resources. | Paid attendance, protected scheduling, eligibility for freelance and contract interpreters, confidential access, suitable locations or online provision, and absence of personal financial penalties. Relevant across employment arrangements, with particular importance for freelance, contract, agency, and platform-based interpreters. | S06, S09, S10, S11, S13, S14, S17 |
| D4, ST31. Organizational protection from abusive, threatening, or violent conduct | Policies and operational procedures that prevent, interrupt, and respond to abusive, discriminatory, sexual, threatening, or violent conduct directed at interpreters or encountered during assignments. This factor focuses on active organizational protection, distinct from reporting mechanisms under ST29. | Organizational safety and safeguarding level; provided by employers, agencies, platforms, service institutions, managers, security personnel, and designated safeguarding staff. Protective procedures may limit exposure to harmful conduct, enable prompt intervention, and facilitate appropriate post-incident support. | Conduct policies, pre-assignment risk assessment, authority to terminate unsafe encounters, removal from abusive calls, security procedures, managerial intervention, legal escalation, reassignment, and post-incident support. Relevant across client-facing contexts, especially remote and video-relay services, healthcare, legal, public-service, conflict-related, crisis, and other high-risk settings. | S07, S12, S18, S19, S21 |

***Note.*** Source IDs correspond to the publications listed in Table S1. The mappings indicate conceptual or empirical support for candidate generation and refinement; they provide no evidence of intervention effectiveness or causal effects. S02 informed the resource-based organization of the four higher-order dimensions and is omitted from the item-level rows to avoid repetition. ST21 and ST23 were presented to the expert panel as composite statements. Their source mappings indicate literature support for the constituent concepts without identifying which component primarily influenced the experts’ ratings. The ecological levels, providers, proposed mechanisms, operationalizations, and relevant contexts or modalities describe the intended conceptual scope of each candidate and require further empirical evaluation.

# ***Table S3.*** Changes from the draft framework to the refined preliminary framework.

| Draft framework item | Refined preliminary framework item | Type of change | Documented rationale and literature alignment |
| --- | --- | --- | --- |
| D1. Individual awareness, regulation, and recovery | D1. Individual awareness, regulation, and recovery | Retained unchanged | The label continued to represent awareness, in-assignment regulation, post-assignment processing, recovery, detachment, standards, and help-seeking. |
| D2. Professional preparedness, agency, and boundary management | D2. Professional preparedness, agency, and sustainable competence | Dimension renamed | “Sustainable competence” broadened the dimension to include continuing professional development and career-long maintenance of competence, while role and boundary management remained represented in ST11. Alignment: S03, S04, S06, S13, S19, and S26. |
| D3. Relational and formal support systems | D3. Collegial, interdisciplinary, and professional support | Dimension renamed | The revised label specifies the three principal sources of support represented by the sub-themes: interpreter colleagues, service-team professionals, and qualified supervisory or psychological providers. Alignment: S09, S11, S12, S14, and S17. |
| D4. Organizational conditions, occupational health protection, and career sustainability | D4. Organizational, employment, and occupational health support | Dimension renamed | The revised label gives clearer attention to employment conditions and organizational responsibility. Career sustainability was treated as a possible outcome of an effective support framework. Alignment: S02, S05, S07, S10, S13, S18, and S19. |
| ST1. Recognition of occupational strain and early warning signs | ST1. Recognition of occupational strain and early warning signs | Retained unchanged | The wording already identified the recognition function and its preventive timing clearly. Alignment: S01, S03, S06, S09, S14, and S15. |
| ST2. Emotional self-regulation during interpreting assignments | ST2. Emotional self-regulation during assignments | Wording shortened | “Interpreting” was removed for concision since the professional context was already established. The in-assignment temporal boundary was retained. Alignment: S03, S06, S16, and S17. |
| ST3. Constructive emotional processing after difficult assignments | ST3. Individual reflection and emotional processing after difficult assignments | Conceptual boundary clarified | “Individual reflection” specifies that the item concerns self-directed processing and distinguishes it from formal post-assignment debriefing under refined ST19. Alignment: S03, S09, S16, and S17. |
| ST4. Restorative physical and psychological self-care routines | ST4. Physical recovery and restorative self-care | Scope clarified | The revised wording emphasizes restoration and physical recovery. Psychological disengagement and work-life boundaries are addressed separately under ST5. Alignment: S01, S03, S06, S15, and S17. |
| ST5. Psychological detachment from work during non-work time | ST5. Psychological detachment and work-life boundaries | Scope expanded | The refined item covers both mental disengagement and the practical maintenance of boundaries between professional and personal life. Alignment: S03, S06, S09, S15, S17, and S24. |
| ST6. Adaptive professional standards and constructive responses to mistakes | ST6. Realistic professional standards and constructive management of mistakes | Concept clarified | “Realistic” makes the relationship with perfectionistic standards more explicit, while “management” emphasizes an active and constructive response to errors. Alignment: S03 and S23. |
| ST7. Timely help-seeking when personal coping is insufficient | ST7. Timely help-seeking when personal coping is insufficient | Retained unchanged | The wording clearly identifies the condition for escalation from self-management to external support. Alignment: S01, S03, S06, S09, S14, S15, S17, and S21. |
| ST8. Assignment-specific contextual and terminological preparation | ST8. Assignment-specific contextual and terminological preparation | Retained unchanged | The item already distinguished assignment-specific preparation from general professional competence. Alignment: S09, S11, S13, S17, and S25. |
| ST9. Competence for the interpreting setting and modality | ST9. Setting- and modality-specific professional competence | Wording clarified | The refined wording identifies professional competence as specific to the setting and modality and improves grammatical precision. Alignment: S01, S08, S09, S13, S17, S19, and S25. |
| ST10. Trauma-informed and emotionally informed interpreting competence | ST10. Trauma- and emotion-informed interpreting competence | Wording condensed | The two related descriptors were expressed more concisely while preserving trauma-related and emotional competence as distinct elements. Alignment: S03, S09, S11, S12, S14, S17, and S21. |
| ST11. Clarity of the interpreter’s professional role and ethical boundaries | ST11. Role clarity and ethical boundary management | Active management specified | The refined item extends knowledge of role and boundaries to their active management during professional practice. Alignment: S09, S11, S12, S16, S17, S21, and S25. |
| ST12. Professional decision latitude in managing assignment demands | ST12. Decision latitude, including the ability to pause, seek assistance, or decline unsafe conditions | Operational meaning added | Examples were added to clarify the professional actions represented by decision latitude and to reduce variation in interpretation by questionnaire respondents. Alignment: S03, S09, S17, and S22. |
| Original D4, ST30. Continuing professional development across the career span | D2, ST13. Continuing professional development across the career span | Moved and renumbered | The item was moved from the organizational dimension to the professional dimension since its primary function is maintenance of professional competence. Organizational responsibility for protected access remains represented under ST30. Alignment: S03, S04, S06, S13, S19, and S26. |
| Original D3, ST13. Peer consultation and mutual support among interpreters | D3, ST14. Peer consultation and mutual support | Wording shortened and item renumbered | “Among interpreters” was removed since the collegial dimension and the term “peer” already establish the provider. Alignment: S03, S06, S09, S11, S14, S15, S17, and S22. |
| Original D3, ST14. Professional mentoring by experienced interpreters | D3, ST15. Professional mentoring | Wording shortened and item renumbered | The definition of mentoring already implies developmental guidance from a more experienced professional. Alignment: S03, S06, and S17. |
| Original D3, ST15. Inclusion of interpreters as members of the service team | D3, ST16. Inclusion as a member of the service team | Wording shortened and item renumbered | The refined wording improves concision while preserving the focus on continuing team membership. Alignment: S11, S12, S16, and S17. |
| Original D3, ST16. Collaborative pre-assignment briefing and case coordination | D3, ST17. Collaborative briefing and case coordination | Temporal scope broadened and item renumbered | Removal of “pre-assignment” allows coordination to occur before or during an encounter while maintaining assignment-specific collaboration as the central concept. Alignment: S03, S09, S11, S12, and S16. |
| Original D3, ST17. Routine confidential professional supervision | D3, ST18. Confidential professional supervision | Wording simplified and item renumbered | “Routine” was removed to avoid prescribing a fixed frequency. Recurrence is retained in the working definition of professional supervision. Alignment: S03, S09, S10, S13, S14, and S22. |
| Original D3, ST18. Structured post-assignment debriefing and case reflection | D3, ST19. Formal post-assignment debriefing | Concept narrowed and item renumbered | “Case reflection” was removed to separate formal facilitated debriefing from self-directed reflection under ST3 and ongoing supervision under ST18. Alignment: S03, S09, S11, S14, S17, S21, and S25. |
| Original D3, ST19. Access to professional counseling and trauma-related psychological care | D3, ST20. Access to qualified psychological and trauma-related care | Provider qualification clarified and item renumbered | “Qualified” emphasizes that clinical or trauma-related support should be provided by appropriately trained professionals. The broader term “psychological care” accommodates different service arrangements. Alignment: S01, S03, S09, S11, S12, S17, S21, and S25. |
| Original D4, ST20. Sustainable workload, scheduling, breaks, and assignment rotation | D4, ST21. Manageable workload, scheduling, breaks, recovery time, and assignment rotation | Scope expanded and item renumbered | “Manageable” provides a clearer evaluative standard, and “recovery time” makes the post-demand recovery period explicit. Alignment: S03, S05, S06, S08-S10, S13, S17, S22, and S26. |
| Original D4, ST21. Hearing protection and acoustic safety | D4, ST22. Acoustic and hearing safety | Wording streamlined and item renumbered | The revised wording places the environmental exposure before its health consequence and avoids implying that personal protective action is the sole response. Alignment: S03, S06, and S19. |
| Original D4, ST22. Vocal health protection; original D4, ST23. Musculoskeletal and repetitive-strain prevention | D4, ST23. Vocal and musculoskeletal health protection | Two candidates merged and renumbered | The two bodily occupational-health factors were merged to reduce fragmentation while retaining voice-related and upper-body risks within the item. Alignment: S03, S18, and S19. |
| Original D4, ST24. Ergonomically and environmentally safe interpreting workspaces | D4, ST24. Ergonomically and environmentally safe workspaces | Wording shortened | “Interpreting” was removed since the framework context already establishes the type of workspace. Alignment: S03, S06, S18, and S19. |
| No corresponding draft item | D4, ST25. Access to reliable technology and technical support | New candidate added | The item was added to represent equipment, platform, connectivity, and troubleshooting requirements in remote and technology-mediated interpreting. Alignment: S05-S07, S17, and S19. |
| Original D4, ST25. Fair remuneration for interpreting and related professional time | D4, ST26. Fair remuneration, including preparation and related professional time | Scope made explicit and item renumbered | “Including preparation” specifies an important category of professional time that may fall outside the interpreted encounter. Alignment: S05, S09, S10, S13, and S19. |
| Original D4, ST26. Employment security and fair contracting conditions | D4, ST27. Employment security and fair contracting | Wording shortened and item renumbered | “Conditions” was removed since it is already implicit in employment security and contracting. Alignment: S05, S06, S09, S10, S13, and S19. |
| Original D4, ST27. Professional recognition, respect, and status | D4, ST28. Professional recognition, respect, and status | Retained and renumbered | The content and wording were retained as a distinct organizational resource. Alignment: S05, S09-S13, and S19. |
| Original D4, ST28. Psychologically safe reporting of overload, errors, and health concerns | D4, ST29. Safe reporting and effective responses to overload, adverse incidents, discrimination, and health concerns | Scope and organizational response expanded | The refined item includes adverse incidents and discrimination and specifies that reporting should lead to an effective organizational response. Alignment: S07, S11-S13, S18, S20, and S21. |
| Original D4, ST29. Equitable, paid, and protected access to organizational support resources | D4, ST30. Protected and remunerated access to organizational support resources | Wording refined and item renumbered | “Remunerated” provides more precise professional wording for paid access. The item focuses on protected time and absence of personal financial loss when using support resources. Alignment: S06, S09-S11, S13-S14, and S17. |
| No corresponding draft item | D4, ST31. Organizational protection from abusive, threatening, or violent conduct | New candidate added | The item was added to address abusive caller or client behavior, discrimination, sexual or racial harassment, threats, and workplace violence as organizational occupational-health responsibilities. Alignment: S07, S12, S18, S19, and S21. |
| Original D4, ST31. Professional meaning, compassion satisfaction, and career continuity | Removed from the refined candidate-factor set | Candidate removed and reclassified | Professional meaning, compassion satisfaction, and career continuity were classified as possible outcomes of supportive working conditions instead of support provisions themselves. Relevant outcome literature remains part of the background corpus. Alignment: S04, S08, S12, S20, and S26. |

***Note.*** Table S3 was compiled during revision by comparing the draft framework in Table 1, the refined preliminary framework in Table 2, and the manuscript’s account of content and face review. It provides a retrospective, document-based account of the recorded changes and does not imply that a contemporaneous change-log table formed part of the original submission. Source IDs in this table identify selected publications most directly aligned with each recorded change; complete source mappings for the refined candidates are provided in Table S2. The wording and placement decisions arose from the content and face review.

# ***Table S4.*** Refined preliminary framework for sustaining interpreters’ well-being and professional functioning.

| Dimension | Candidate sub-theme | Operational definition in the present study | Supporting evidence (source IDs) |
| --- | --- | --- | --- |
| D1. Individual awareness, regulation, and recovery | ST1. Recognition of occupational strain and early warning signs | Awareness of cognitive, emotional, behavioral, or physical indicators that occupational demands are becoming difficult to manage and may require recovery or further support. | S01, S09, S14 |
|  | ST2. Emotional self-regulation during assignments | Strategies used during an assignment to regulate arousal and emotional responses while maintaining attention and performing the interpreting task. | S06, S16, S17 |
|  | ST3. Individual reflection and emotional processing after difficult assignments | Self-directed reflection after an emotionally difficult assignment to examine personal reactions, process the experience, and identify any need for further support. It is distinguished from formally provided debriefing under ST19. | S03, S09, S17 |
|  | ST4. Physical recovery and restorative self-care | Personally undertaken restorative activities that address fatigue and support physical and psychological recovery, including adequate rest, sleep, movement, and other appropriate self-care practices. | S01, S06, S15 |
|  | ST5. Psychological detachment and work–life boundaries | Mental disengagement from interpreting work during non-work time and maintenance of boundaries between professional responsibilities and personal life. | S06, S17, S24 |
|  | ST6. Realistic professional standards and constructive management of mistakes | Use of attainable professional standards, proportionate self-evaluation, and constructive responses to errors or perceived shortcomings. | S03, S23 |
|  | ST7. Timely help-seeking when personal coping is insufficient | Recognition of the limits of personal coping and timely use of appropriate peer, supervisory, organizational, or professional assistance. | S01, S14, S17 |
| D2. Professional preparedness, agency, and sustainable competence | ST8. Assignment-specific contextual and terminological preparation | Preparation for the subject matter, terminology, participants, purpose, communicative context, and foreseeable demands of a particular assignment. | S09, S11, S17, S25 |
|  | ST9. Setting- and modality-specific professional competence | Knowledge and skills appropriate to the professional setting, interpreting modality, communication format, and needs of the participants involved. | S09, S19, S25 |
|  | ST10. Trauma- and emotion-informed interpreting competence | Knowledge and professional practices for recognizing trauma-related or emotionally intense content and managing its implications for interpreting conduct and personal well-being. | S14, S17, S21 |
|  | ST11. Role clarity and ethical boundary management | Understanding the interpreter’s responsibilities and professional limits and applying ethical boundaries in interactions, relationships, and professional decision-making. | S09, S11, S16 |
|  | ST12. Decision latitude, including the ability to pause, seek assistance, or decline unsafe conditions | Professional discretion to pause an assignment, request clarification or assistance, seek changes to unsuitable conditions, or decline work considered unsafe or beyond the interpreter’s competence. | S03, S17, S22 |
|  | ST13. Continuing professional development across the career span | Ongoing learning, training, reflection, and skill renewal used to maintain or extend professional competence throughout an interpreting career. | S06, S13, S26 |
| D3. Collegial, interdisciplinary, and professional support | ST14. Peer consultation and mutual support | Reciprocal exchange among interpreters for practical advice, shared reflection, professional validation, and emotional or collegial support. | S06, S09, S17 |
|  | ST15. Professional mentoring | Developmental guidance provided over time by a more experienced interpreter or practitioner to support professional learning, decision-making, and adjustment to occupational demands. | S03, S06, S17 |
|  | ST16. Inclusion as a member of the service team | Recognition and appropriate integration of the interpreter into the service team’s communication, information exchange, and working processes. | S11, S12, S17 |
|  | ST17. Collaborative briefing and case coordination | Assignment-specific exchange of relevant information and coordination of roles, expectations, sensitive content, risks, and procedures with other professionals before and, where needed, during an assignment. | S09, S11, S12 |
|  | ST18. Confidential professional supervision | Regular and confidential discussion of professional experiences with a suitably qualified supervisor for structured reflection, professional learning, and support. | S09, S10, S14 |
|  | ST19. Formal post-assignment debriefing | A structured and assignment-specific opportunity following a demanding event to review the case, professional decisions, team processes, and emotional responses. | S11, S17, S25 |
|  | ST20. Access to qualified psychological and trauma-related care | Availability of assessment, counseling, or other appropriate care from qualified mental-health professionals when distress or trauma-related effects require specialist attention. | S01, S11, S17 |
| D4. Organizational, employment, and occupational health support | ST21. Manageable workload, scheduling, breaks, recovery time, and assignment rotation | Organizational work-design arrangements governing workload volume and intensity, scheduling, protected breaks, recovery time, and rotation across demanding assignments. | S08, S09, S13, S17 |
|  | ST22. Acoustic and hearing safety | Measures addressing potentially harmful sound exposure, unsuitable listening conditions, and equipment-related acoustic risks in interpreting work. | S03, S06, S19 |
|  | ST23. Vocal and musculoskeletal health protection | Measures addressing vocal strain and musculoskeletal or repetitive-strain risks associated with the physical performance of interpreting tasks. | S03, S18, S19 |
|  | ST24. Ergonomically and environmentally safe workspaces | Physical and environmental working conditions that support safe posture, adequate visibility and audibility, concentration, mobility, and physical comfort. | S06, S18, S19 |
|  | ST25. Access to reliable technology and technical support | Availability of dependable platforms, devices, connectivity, and timely technical assistance for remote, video-mediated, or otherwise technology-supported interpreting. | S05, S06, S07, S19 |
|  | ST26. Fair remuneration, including preparation and related professional time | Compensation that covers the interpreting assignment and associated professional activities, including preparation, briefing, debriefing, waiting, and other required work time. | S05, S09, S10, S13 |
|  | ST27. Employment security and fair contracting | Stable, transparent, and equitable employment or contractual conditions, including clarity concerning duties, payment, continuity of work, and applicable protections. | S05, S06, S13 |
|  | ST28. Professional recognition, respect, and status | Recognition of interpreting as skilled professional work, expressed through respectful treatment, appropriate professional status, and acknowledgment of interpreters’ expertise and contribution. | S09, S10, S11, S13 |
|  | ST29. Safe reporting and effective responses to overload, adverse incidents, discrimination, and health concerns | Confidential, accessible, and non-retaliatory channels for reporting occupational concerns, accompanied by timely organizational review, follow-up, and appropriate action. | S07, S13, S18 |
|  | ST30. Protected and remunerated access to organizational support resources | Access to supervision, training, psychological support, or related organizational resources during formally protected and remunerated professional time. | S09, S11, S13, S17 |
|  | ST31. Organizational protection from abusive, threatening, or violent conduct | Policies and procedures designed to prevent, report, investigate, and respond to abusive, threatening, discriminatory, or violent conduct directed at interpreters. | S07, S18, S21 |

***Note.*** Operational definitions delimit the intended scope of the candidate sub-themes in the present study. Supporting-evidence codes refer to the publications listed in Table S1 and identify literature that informed the development or refinement of each candidate. These codes are illustrative and should be read with the fuller source mapping in Table S2. The mapping records conceptual or empirical support for candidate inclusion; causal effects and intervention effectiveness were outside its scope. Composite candidates, including ST21 and ST23, group related provisions, and the cited publications may address individual components or the broader occupational mechanism. Table S4 records the 31-item refined preliminary framework evaluated by the expert panel. Subsequent FDM analysis retained 27 candidates.

# ***Table S5.*** FDM questionnaire used for expert evaluation: background-information items and seven-point importance-rating sections.

| **Background-information section**  **Instruction:** *Please select one response for each categorical item and enter your age in completed years for B2. For professional role, select the role that best represents your principal professional involvement in interpreting.* | | | | | | | | | |
| --- | --- | --- | --- | --- | --- | --- | --- | --- | --- |
| Item | Background variable | Response options | | | | | | | |
| B1 | Gender | ○ Male   ○ Female | | | | | | | |
| B2 | Age | years | | | | | | | |
| B3 | Highest academic qualification | ○ Master’s degree  ○ Doctoral degree | | | | | | | |
| B4 | Principal professional role | ○ Practicing interpreter   ○ Interpreting educator (Lecturer)  ○ Interpreting educator (Associate Professor)  ○ Interpreting educator (Professor) ○ Interpreting trainer (Lecturer)  ○ Interpreting trainer (Senior Lecturer)  ○ Interpreting researcher  ○ Manager of interpreting services | | | | | | | |
| B5 | Relevant professional experience | ○ 10 years or less   ○ 11–20 years   ○ 21–30 years  ○ 31 years or more | | | | | | | |
| **Section 1. Importance ratings for the proposed dimensions**  **Instruction:** *Please rate the importance of each proposed dimension for sustaining interpreters’ well-being and professional functioning.*  Response scale: 1 = extremely unimportant; 2 = unimportant; 3 = somewhat unimportant; 4 = neutral; 5 = somewhat important; 6 = important; and 7 = extremely important. Please select one response for each item. | | | | | | | | | |
| Code and dimension | | | 1 | 2 | 3 | 4 | 5 | 6 | 7 |
| D1. Individual awareness, regulation, and recovery | | | ○ | ○ | ○ | ○ | ○ | ○ | ○ |
| D2. Professional preparedness, agency, and sustainable competence | | | ○ | ○ | ○ | ○ | ○ | ○ | ○ |
| D3. Collegial, interdisciplinary, and professional support | | | ○ | ○ | ○ | ○ | ○ | ○ | ○ |
| D4. Organizational, employment, and occupational health support | | | ○ | ○ | ○ | ○ | ○ | ○ | ○ |
| **Section 2. Importance ratings for the candidate sub-themes**  **Instruction:** *Please rate the importance of each candidate sub-theme for sustaining interpreters’ well-being and professional functioning.*  Response scale: 1 = extremely unimportant; 2 = unimportant; 3 = somewhat unimportant; 4 = neutral; 5 = somewhat important; 6 = important; and 7 = extremely important. Please select one response for each item. | | | | | | | | | |
| **D1. Individual awareness, regulation, and recovery** | | | | | | | | | |
| **Code and candidate sub-theme** | | | 1 | 2 | 3 | 4 | 5 | 6 | 7 |
| ST1. Recognition of occupational strain and early warning signs | | | ○ | ○ | ○ | ○ | ○ | ○ | ○ |
| ST2. Emotional self-regulation during assignments | | | ○ | ○ | ○ | ○ | ○ | ○ | ○ |
| ST3. Individual reflection and emotional processing after difficult assignments | | | ○ | ○ | ○ | ○ | ○ | ○ | ○ |
| ST4. Physical recovery and restorative self-care | | | ○ | ○ | ○ | ○ | ○ | ○ | ○ |
| ST5. Psychological detachment and work–life boundaries | | | ○ | ○ | ○ | ○ | ○ | ○ | ○ |
| ST6. Realistic professional standards and constructive management of mistakes | | | ○ | ○ | ○ | ○ | ○ | ○ | ○ |
| ST7. Timely help-seeking when personal coping is insufficient | | | ○ | ○ | ○ | ○ | ○ | ○ | ○ |
| **D2. Professional preparedness, agency, and sustainable competence** | | | | | | | | | |
| **Code and candidate sub-theme** | | | 1 | 2 | 3 | 4 | 5 | 6 | 7 |
| ST8. Assignment-specific contextual and terminological preparation | | | ○ | ○ | ○ | ○ | ○ | ○ | ○ |
| ST9. Setting- and modality-specific professional competence | | | ○ | ○ | ○ | ○ | ○ | ○ | ○ |
| ST10. Trauma- and emotion-informed interpreting competence | | | ○ | ○ | ○ | ○ | ○ | ○ | ○ |
| ST11. Role clarity and ethical boundary management | | | ○ | ○ | ○ | ○ | ○ | ○ | ○ |
| ST12. Decision latitude, including the ability to pause, seek assistance, or decline unsafe conditions | | | ○ | ○ | ○ | ○ | ○ | ○ | ○ |
| ST13. Continuing professional development across the career span | | | ○ | ○ | ○ | ○ | ○ | ○ | ○ |
| **D3. Collegial, interdisciplinary, and professional support** | | | | | | | | | |
| **Code and candidate sub-theme** | | | 1 | 2 | 3 | 4 | 5 | 6 | 7 |
| ST14. Peer consultation and mutual support | | | ○ | ○ | ○ | ○ | ○ | ○ | ○ |
| ST15. Professional mentoring | | | ○ | ○ | ○ | ○ | ○ | ○ | ○ |
| ST16. Inclusion as a member of the service team | | | ○ | ○ | ○ | ○ | ○ | ○ | ○ |
| ST17. Collaborative briefing and case coordination | | | ○ | ○ | ○ | ○ | ○ | ○ | ○ |
| ST18. Confidential professional supervision | | | ○ | ○ | ○ | ○ | ○ | ○ | ○ |
| ST19. Formal post-assignment debriefing | | | ○ | ○ | ○ | ○ | ○ | ○ | ○ |
| ST20. Access to qualified psychological and trauma-related care | | | ○ | ○ | ○ | ○ | ○ | ○ | ○ |
| **D4. Organizational, employment, and occupational health support** | | | | | | | | | |
| **Code and candidate sub-theme** | | | 1 | 2 | 3 | 4 | 5 | 6 | 7 |
| ST21. Manageable workload, scheduling, breaks, recovery time, and assignment rotation | | | ○ | ○ | ○ | ○ | ○ | ○ | ○ |
| ST22. Acoustic and hearing safety | | | ○ | ○ | ○ | ○ | ○ | ○ | ○ |
| ST23. Vocal and musculoskeletal health protection | | | ○ | ○ | ○ | ○ | ○ | ○ | ○ |
| ST24. Ergonomically and environmentally safe workspaces | | | ○ | ○ | ○ | ○ | ○ | ○ | ○ |
| ST25. Access to reliable technology and technical support | | | ○ | ○ | ○ | ○ | ○ | ○ | ○ |
| ST26. Fair remuneration, including preparation and related professional time | | | ○ | ○ | ○ | ○ | ○ | ○ | ○ |
| ST27. Employment security and fair contracting | | | ○ | ○ | ○ | ○ | ○ | ○ | ○ |
| ST28. Professional recognition, respect, and status | | | ○ | ○ | ○ | ○ | ○ | ○ | ○ |
| ST29. Safe reporting and effective responses to overload, adverse incidents, discrimination, and health concerns | | | ○ | ○ | ○ | ○ | ○ | ○ | ○ |
| ST30. Protected and remunerated access to organizational support resources | | | ○ | ○ | ○ | ○ | ○ | ○ | ○ |
| ST31. Organizational protection from abusive, threatening, or violent conduct | | | ○ | ○ | ○ | ○ | ○ | ○ | ○ |

***Note.*** The same seven-point importance scale was used for all four dimension items and all 31 candidate sub-theme items. One response was provided for each item; age was entered in completed years, and one option was selected for each categorical item. The table reproduces the questionnaire content following the study-information and electronic-consent screen.

# **References**

Alhawamdeh, S., & Zhang, C. (2021). Wellness of interpreters: Stress-related occupational hazards and possible solutions. New Voices in Translation Studies, 24(1), 82–93. <https://doi.org/10.14456/nvts.2021.6>

Bakker, A. B., & Demerouti, E. (2017). Job demands–resources theory: Taking stock and looking forward. Journal of Occupational Health Psychology, 22(3), 273–285. <https://doi.org/10.1037/ocp0000056>

Brunale, L., & Sannolla, A. (2026). Wellbeing promoting practices for interpreters. In G. Mack & A. Amato (Eds.), Interpreters’ health and wellbeing: An overview across modes, modalities and settings (pp. 231–256). Routledge. <https://doi.org/10.4324/9781003586647-15>

Chen, T. (2023). The interplay between psychological well-being, stress, and burnout: Implications for translators and interpreters. Heliyon, 9(8), Article e18589. <https://doi.org/10.1016/j.heliyon.2023.e18589>

Courtney, J., & Phelan, M. (2019). Translators’ experiences of occupational stress and job satisfaction. Translation & Interpreting, 11(1), 100–113. <https://doi.org/10.12807/ti.111201.2019.a06>

Crezee, I., & Lai, M. (2022). Interpreters’ resilience and self-care during pandemic restrictions in Australia and New Zealand. New Voices in Translation Studies, 27(1), 90–118. <https://doi.org/10.14456/nvts.2022.23>

Dean, R. K., Cerulli, C., Devor, D. J., Pollard, R. Q., Jr., Biello, S. E., Maffia, D., & Crean, H. F. (2025). Video Relay Service interpreters’ experiences with caller behavior: An occupational health risk call to action. Healthcare, 13(17), Article 2116. <https://doi.org/10.3390/healthcare13172116>

Geiling, A., Böttche, M., Knaevelsrud, C., & Stammel, N. (2022). A comparison of interpreters’ wellbeing and work-related characteristics in the care of refugees across different work settings. BMC Public Health, 22, Article 1635. <https://doi.org/10.1186/s12889-022-14034-7>

Geiling, A., Knaevelsrud, C., Böttche, M., & Stammel, N. (2021). Mental health and work experiences of interpreters in the mental health care of refugees: A systematic review. Frontiers in Psychiatry, 12, Article 710789. <https://doi.org/10.3389/fpsyt.2021.710789>

Geiling, A., Knaevelsrud, C., Böttche, M., & Stammel, N. (2022). Psychological distress, exhaustion, and work-related correlates among interpreters working in refugee care: Results of a nationwide online survey in Germany. European Journal of Psychotraumatology, 13(1), Article 2046954. <https://doi.org/10.1080/20008198.2022.2046954>

Goldhirsch, J., Scott, N., Ma, Y., & Abrahm, J. (2022). Addressing vicarious trauma and emotional stress among medical interpreters through care, partnership and respect. New Voices in Translation Studies, 27(1), 119–141. <https://doi.org/10.14456/nvts.2022.24>

Graves, J. M., Ross, N., Gonzalez, C., Moore, M., Denson, V., & Vavilala, M. S. (2023). Compassion and trauma: Occupational health experiences of certified medical interpreters in five U.S. states. Workplace Health & Safety, 71(9), 436–444. <https://doi.org/10.1177/21650799231167586>

Hanft-Robert, S., & Mösko, M. (2024). Community interpreting in Germany: Results of a nationwide cross-sectional study among interpreters. BMC Public Health, 24, Article 1570. <https://doi.org/10.1186/s12889-024-18988-8>

Hapfelmeier, G., Walfisch, L., Schroers, L., Bender, S., & Walg, M. (2025). Secondary traumatic stress in interpreters for refugees: Why training and supervision matter. Psychiatry International, 6(3), Article 91. <https://doi.org/10.3390/psychiatryint6030091>

Korpal, P., & Mellinger, C. D. (2022). Self-care strategies of professional community interpreters: An interview-based study. Translation, Cognition & Behavior, 5(2), 275–299. <https://doi.org/10.1075/tcb.00069.kor>

Leanza, Y., Pointurier, S., & Duchesne, L. (2025). Interpreting is an emotional effort: Public service interpreters’ coping strategies in emotionally charged situations. Interpreting and Society: An Interdisciplinary Journal, 5(1), 3–26. <https://doi.org/10.1177/27523810241310909>

Lim, P. S., Olen, A., Carballido, J. K., LiaBraaten, B. M., Sinnen, S. R., Balistreri, K. A., Tager, J. B., Rothschild, C. B., Scanlon, M. C., Davies, W. H., & Nordness, K. (2022). “We need a little help”: A qualitative study on distress and coping among pediatric medical interpreters. Journal of Hospital Management and Health Policy, 6, Article 36. <https://doi.org/10.21037/jhmhp-22-23>

Louzán, R., Cuervo-Carabel, T., Ortiz-López, M., & Gómez Armesto, L. (2025). Ergonomic and psychosocial risks in sign language interpreters and guide-interpreters: A systematic review and bibliometric analysis. Ergonomics. Advance online publication. <https://doi.org/10.1080/00140139.2025.2519871>

Mack, G., & Amato, A. (Eds.). (2026). Interpreters’ health and wellbeing: An overview across modes, modalities and settings. Routledge. <https://doi.org/10.4324/9781003586647>

Palmer, J. L., Winninghoff, J., Boland, A. S., & Bloom, C. L. (2025). Burnout among sign language interpreters working in postsecondary institutions. Interpreting, 27(2), 281–302. <https://doi.org/10.1075/intp.00122.pal>

Polat Ulaş, A. (2022). Emotional challenges of interpreters working with refugees in Türkiye. New Voices in Translation Studies, 27(1), 64–89. <https://doi.org/10.14456/nvts.2022.22>

Schwenke, T. (2012). Sign language interpreters and burnout. Journal of Interpretation, 20(1), Article 7. <https://digitalcommons.unf.edu/joi/vol20/iss1/7/>

Schwenke, T. J., Ashby, J. S., & Gnilka, P. B. (2014). Sign language interpreters and burnout: The effects of perfectionism, perceived stress, and coping resources. Interpreting, 16(2), 209–232. <https://doi.org/10.1075/intp.16.2.04sch>

Sonnentag, S., & Fritz, C. (2007). The Recovery Experience Questionnaire: Development and validation of a measure for assessing recuperation and unwinding from work. Journal of Occupational Health Psychology, 12(3), 204–221. <https://doi.org/10.1037/1076-8998.12.3.204>

Valero-Garcés, C. (2015). The impact of emotional and psychological factors on public service interpreters: Preliminary studies. Translation & Interpreting, 7(3), 90–102. <https://doi.org/10.12807/ti.107203.2015.a07>

Yan, D. A., & Jia, C. (2025). Occupational stress and turnover intentions among simultaneous interpreters: The mediating role of burnout and the moderating role of grit. Perspectives. Advance online publication. https://doi.org/10.1080/0907676X.2025.2478403
